# Predicting early recurrence after resection of initially unresectable colorectal liver metastases: the role of baseline and pre-surgery clinical, radiological and molecular factors in a real-life multicentre experience

**DOI:** 10.1016/j.esmoop.2024.102991

**Published:** 2024-04-16

**Authors:** R. Moretto, M.M. Germani, B. Borelli, V. Conca, D. Rossini, P. Boraschi, F. Donati, L. Urbani, S. Lonardi, F. Bergamo, K. Cerma, G. Ramondo, F.E. D’Amico, L. Salvatore, G. Valente, B. Barbaro, F. Giuliante, M. Di Maio, G. Masi, C. Cremolini

**Affiliations:** 1 Unit of Medical Oncology 2, Azienda Ospedaliero-Universitaria Pisana, Pisa; 2 Department of Translational Research and New Technologies in Medicine, University of Pisa, Pisa; 3 Department of Health Sciences, Section of Clinical Pharmacology and Oncology, University of Florence, Pisa; 4 Department of Diagnostic and Interventional Radiology, and Nuclear Medicine, Azienda Ospedaliero-Universitaria Pisana, Pisa; 5 General Surgery, Azienda Ospedaliero-Universitaria Pisana, Pisa; 6 Department of Oncology, Veneto Institute of Oncology IOV – IRCCS, Padua; 7 Radiology Unit, Veneto Institute of Oncology IOV – IRCCS, Padua; 8 General Surgery 2, Department of Surgical Oncological and Gastroenterological Sciences (DISCOG), University of Padua, Padua; 9 Medical Oncology Unit, Comprehensive Cancer Center, Fondazione Policlinico Universitario Agostino Gemelli IRCCS, Rome; 10 Medical Oncology Unit, Università Cattolica del Sacro Cuore, Rome; 11 Diagnostic and General Interventional Radiology, Fondazione Policlinico Universitario Agostino Gemelli, IRCCS, Rome; 12 General and Hepatobiliary Surgery, Fondazione Policlinico Universitario Agostino Gemelli, IRCCS, Rome; 13 Department of Oncology, Università degli Studi di Torino, Turin, Italy

**Keywords:** unresectable colorectal liver metastases, secondary resection, conversion chemotherapy, early disease recurrence

## Abstract

**Background:**

Advances in surgical techniques and systemic treatments have increased the likelihood of achieving radical surgery and long-term survival in metastatic colorectal cancer (mCRC) patients with initially unresectable colorectal liver metastases (CRLMs). Nonetheless, roughly half of the patients resected after an upfront systemic therapy experience disease relapse within 6 months from surgery, thus leading to the question whether surgery is actually beneficial for these patients.

**Materials and methods:**

A real-world dataset of mCRC patients with initially unresectable liver-limited disease treated with conversion chemotherapy followed by radical resection of CRLMs at three high-volume Italian institutions was retrospectively assessed with the aim of investigating the association of baseline and pre-surgical clinical, radiological and molecular factors with the risk of relapse within 6 or 12 months from surgery.

**Results:**

Overall, 268 patients were included in the analysis and 207 (77%) experienced recurrence. Ninety-six (46%) of them had disease relapse within 6 months after CRLM resection and in spite of several variables associated with early recurrence at univariate analyses, only primary tumour resection at diagnosis [odds ratio (OR) 0.53, 95% confidence interval (CI) 0.32-0.89, *P* = 0.02] remained significant in the multivariable model. Among patients with resected primary tumours, pN+ stage was associated with higher risk of disease relapse within 6 months (OR 3.02, 95% CI 1.23-7.41, *P* = 0.02). One hundred and forty-nine patients (72%) had disease relapse within 12 months after CRLMs resection but none of the analysed variables was independently associated with outcome.

**Conclusions:**

Clinical, radiological and molecular factors assessed before and after conversion chemotherapy do not reliably predict early recurrence after secondary resection of initially unresectable CRLMs. While novel markers are needed to optimize the cost/efficacy balance of surgical procedures, CRLM resection should be offered as soon as metastases become resectable during first-line chemotherapy to all patients eligible for surgery.

## Highlights

• Early relapse (<6 months) after resection of colorectal liver metastases occurs in 35%-45% of liver-limited mCRC patients.
• Predictors of early relapse are needed to propose surgery to patients turned resectable after conversion chemotherapy.
• In this retrospective real-world analysis, no reliable biomarkers were found to build an accurate prognostic model.
• Novel biomarkers (i.e. ctDNA and radiomic features) are awaited to refine surgical selection after upfront chemotherapy.

## Introduction

Metastatic colorectal cancer (mCRC) is a fatal disease in the vast majority of cases. However, patients with colorectal liver metastases (CRLMs) may be eligible for metastases resection achieving long-term disease remission and survival.^1^ Liver-limited disease occurs in around 20%-30% of mCRC patients, and 10%-20% of them are deemed upfront resectable while 30%-40% are initially unresectable but potentially amenable for surgery if tumour shrinkage is achieved by conversion chemotherapy.^2^^,^^3^ Recent advances in surgical techniques, the availability of increasingly active systemic treatments and the widespread of application the multidisciplinary approach have expanded the percentage of patients with CRLMs deemed eligible for radical surgery.^4^, ^5^, ^6^, ^7^ However, although 20%-30% of resected patients can achieve a long-term overall survival benefit from liver metastasectomy,^8^ most patients relapse during the first 2 years after hepatectomy,^9^^,^^10^ with about 35%-45% of early recurrences occurring within 6 months after surgery.^6^^,^^11^ It is not clear whether in these cases liver resection may still have a favourable impact on the subsequent steps of disease history and ultimately on patients’ survival. At the same time, liver resection is encumbered by non-neglectable perioperative mortality and severe morbidity rates around 1%-3% and 30%, respectively.^12^ In order to maximize the risk/benefit balance of liver surgery and to avoid futile procedures, both technical/surgical and oncological/prognostic criteria should be considered.^1^^,^^13^ Although a large number of prognostic scores including clinical, pathological and molecular parameters have been proposed, most of them focused on patients eligible for upfront liver metastasectomy and were developed in series where suboptimal systemic regimens were adopted.^14^, ^15^, ^16^, ^17^, ^18^, ^19^ Moreover, the impact of individual variables on the risk of early recurrence was not investigated, and potentially prognostic variables were never evaluated after the systemic therapy.^11^

Drawing from these considerations, we assessed the risk of early recurrence according to baseline and pre-surgery clinical, radiological and molecular parameters in patients with initially unresectable liver-limited CRLMs undergoing resection after first-line chemotherapy, with the aim of building a reliable prognostic model that may support clinicians to offer liver surgery after initial systemic treatment.

## Materials and methods

### Study population

Consecutive patients with resected CRLMs referred to three Italian high-volume institutions with expertise in liver surgery (Azienda Ospedaliero-Universitaria Pisana, Pisa; Veneto Institute of Oncology, Padua; Fondazione Policlinico Universitario Agostino Gemelli IRCCS, Roma) from January 2009 to January 2022 were selected. Eligibility criteria were: R0/R1 resection of liver metastases and primary tumour if not yet resected at the time of the beginning of conversion chemotherapy; absence of extrahepatic disease at the time of CRLM diagnosis; CRLMs deemed initially unresectable for technical and/or oncological reasons as for local multidisciplinary assessment [more than five liver metastases,^20^ maximum size of the larger metastasis ≥5 cm, *BRAFV600E* mutated and technically difficult to resect; R0 resection possible with complex minor hepatectomy,^21^ portal vein embolization, two-stage hepatectomy, hepatectomy combined with ablation, associating liver partition and portal vein ligation for staged hepatectomy (ALPPS), complex major hepatectomy^1^^,^^13^^,^^22^^,^^23^]; administration of first-line treatment before liver surgery, including doublet (FOLFOX: 5-fluorouracil and oxaliplatin; CAPOX: capecitabine and oxaliplatin; FOLFIRI: 5-Fluorouracil and irinotecan) or triplet (FOLFOXIRI: 5-fluorouracil, oxaliplatin and irinotecan) plus or minus anti-vascular endothelial growth factor (anti-VEGF, bevacizumab)/epidermal growth factor receptor (anti-EGFRs, cetuximab or panitumumab) monoclonal antibodies^1^^,^^13^; no evidence of radiological progression before surgery; availability of baseline and pre-surgery imaging [contrast-enhanced computed tomography and/or liver-specific contrast-enhanced magnetic resonance (MRI)] that were independently reviewed by radiologists with liver imaging expertise at each institution (PB and FMD for Azienda Ospedaliero-Universitaria Pisana; GR for Veneto Institute of Oncology, Padua; BB for Fondazione Policlinico Universitario Agostino Gemelli IRCCS) blinded to clinical information, treatment regimen and outcome in order to accurately evaluate the extent of hepatic disease (number and size of liver lesions, relationship of metastases with major hepatic vessels, number of hepatic segments and lobes involved at baseline and pre-surgery); and a minimum follow-up of at least 12 months after liver surgery. Radiological assessments were scheduled every 2-3 months and multidisciplinary meetings with image review were carried out after each scan in order to offer surgery as soon as the disease became resectable. The study was approved by the ethical review board of the coordinating centre (University of Pisa, ID: 3920/2013) and was conducted in accordance with the ethical principles for medical research involving human subjects adopted in the Declaration of Helsinki.

### Statistical analyses

The primary objective of the present study is to identify baseline and pre-surgery clinical, radiological and molecular factors associated to early recurrence defined as the evidence of radiological disease relapse or death, whichever occurred first, within 6 months from liver surgery. Univariate and multivariate logistic regression analyses were carried out to assess the independent predictive value of clinical, radiological and molecular factors in terms of early relapse. The same analyses were carried out using a 12-month cut-off for the definition of early relapse.

Descriptive statistics were used to summarise clinical, radiological and molecular prognostic characteristics. The changes in variables evaluated before and after systemic therapy (i.e. at baseline and pre-surgery) were analysed by means of Wilcoxon or McNemar tests as appropriate. Disease-free survival (DFS) was defined as the time elapsed from liver surgery to the first radiological evidence of disease relapse or death from any cause, whichever occurred first. Overall survival (OS) was defined as the time elapsed from liver surgery to death from any cause. Post-relapse OS was defined as the time elapsed from recurrence after liver surgery to death from any cause. In case of two-stage hepatectomy or staged resection of liver metastases and primary tumour, DFS and OS were calculated from the date of the last surgical procedure. Survival curves were estimated by the Kaplan–Meier method and compared with the log-rank test. Hazard ratios (HRs) with 95% confidence intervals (CIs) were estimated with a Cox proportional hazards model. The impact of clinical, radiological and molecular prognostic variables on DFS and OS was assessed by means of univariate and multivariate Cox regression analyses.

For both logistic and regression models, two parallel analyses were conducted:

- Overall population, where the pT and pN variables were excluded because they were unknown for patients with unresected primary tumour at baseline.
- Baseline resected primary tumour population, where the pT and pN variables were included.

Statistical significance for univariate and multivariate logistic and Cox regression analyses were set at *P* ≤ 0.10 and *P* ≤ 0.05, respectively. All analyses were carried out with R-Studio version 2022.07.02.

## Results

From a shared multi-institutional dataset including 1033 patients with resected CRLMs, 268 patients met the eligibility criteria and were included in the analysis (Supplementary Figure S1, available at https://doi.org/10.1016/j.esmoop.2024.102991). Baseline characteristics are summarised in Table 1. Most of the patients had Eastern Cooperative Oncology Group performance status (ECOG-PS) of 0 (87%), age <65 years (58%) and presented with synchronous liver metastases (82%) and left-sided primary tumours (72%). About half of the tumours were *RAS* mutated (53%), with a very small percentage of *BRAF* mutated (2%) and almost all were proficient mismatch repair/microsatellite stable (pMMR/MSS) (98%). Primary tumour was resected at baseline in 57% of cases, and most of them were pT1-3 (80%) and with positive lymph nodes (70%).

**Table 1:** Patients’ characteristics

Factors		Baseline (%)	Before CRLM resection (%)	*P* value
Age at the diagnosis of CRLMs (continuous)	Median age (IQR) (range)	62 (52-69) (30-83)	—	—

Age at the diagnosis of CRLMs (dichotomous)	<65 years	156 (58)	—	—
	≥65 years	112 (42)		
Sex	Male	159 (59)	—	—
	Female	109 (41)		
ECOG-PS at the diagnosis of CRLMs	0	220 (87)	—	—
	1	33 (13)		
	2	1 (<1)		
	NA	14		
Primary tumour location	Left colon and rectum	194 (72)	—	—
	Right colon	74 (28)		
CRLM synchronous to primary tumour diagnosis (≤6 months)	Yes	219 (82)	—	—
	No	49 (18)		
Primary tumour resected at diagnosis	Yes	154 (57)	—	—
	No	114 (43)		
pT stage in tumours resected at diagnosis	T4	31 (20)	—	—
	T1-T3	123 (80)		
	Not resected at diagnosis	114		
pN stage in tumours resected at diagnosis	N1-2	108 (70)	—	—
	N0	46 (30)		
	Not resected at diagnosis	114		
*RAS* and *BRAF* status	*RAS* mutation	140 (53)	—	—
	*BRAF* mutation	6 (2)		
	*RAS* and *BRAF* wild-type	118 (45)		
	NA	4		
Mismatch repair/microsatellite status	Proficient/stable	234 (98)	—	—
	Deficient/instable	5 (2)		
	NA	29		
MRI with gadolinium-based contrast	Yes	91 (34)	132 (49)	**0.0001**^a^
	No	177 (66)	136 (51)	
First-line treatment regimen	Triplet	112 (42)	—	—
	Doublets	156 (58)		
First-line targeted therapy	Anti-VEGF	175 (66)	—	—
	Anti-EGFR	68 (25)		
	None	25 (9)		
Duration of first-line chemotherapy	Median (IQR) (range)	3.7 (2.7-5.1) (0.6-30.2)	—	—
Objective response	Yes	—	202 (75)	—
	No	—	66 (25)	—
CEA (continuous) ng/ml	Median value (IQR)	17 (5-104)	4 (2-11)	**<0.0001**^b^
CEA (dichotomous) ng/ml	≥10	95 (43)	53 (30)	**<0.0001**^a^
	<10	127 (57)	123 (70)	
	NA	46	92	
Bilobar involvement	Yes	157 (59)	131 (49)	**<0.001**^a^
	No	111 (41)	137 (51)	
Number of segments involved	≥4	141 (53)	104 (39)	**<0.0001**^a^
	<4	127 (47)	164 (61)	
Number of lesions (continuous)	Median value (IQR)	4 (2-6)	3 (2-5)	**<0.0001**^b^
Number of lesions (dichotomous)	≥4	134 (50)	111 (41)	**<0.0001**^a^
	<4	134 (50)	157 (59)	
Max diameter of the largest lesion (mm)	Median value (IQR)	42 (28-65)	25 (16-42)	**<0.0001**^b^
Lesions in contact with major hepatic vessels	Yes	170 (63)	158 (59)	**0.04**^a^
	No	98 (37)	110 (41)	
Disappearance of at least one CRLM	Yes	—	181 (67)	—
	No		87 (33)	

CEA, carcinoembryonic antigen; CRLM, colorectal liver metastasis; ECOG-PS, Eastern Cooperative Oncology Group performance status; EGFR, epidermal growth factor receptor; IQR, interquartile range; mm, millimetres; MRI, magnetic resonance imaging; NA, not available; VEGF, vascular endothelial growth factor.

a McNemar test.

b Paired-sample Wilcoxon test.

At baseline, liver metastases were more frequently bilobar (59%), involved more than four hepatic segments (53%) and were in contact with at least one major hepatic vessel (63%). The median size of the largest lesion was 42 mm [interquartile range (IQR) 28-65 mm, range 6-205 mm] and the median number of lesions was 4 (IQR 2-6, range 1-64). Carcinoembryonic antigen (CEA) levels were higher than 10 ng/ml in 43% of patients with a median value of 17 ng/ml (IQR 5-104 ng/ml). Baseline MRI was carried out in 34% of patients.

Overall, triplet and doublet chemotherapy were administered in 42% and 58% of patients, respectively, with the addition of a biologic agent in 91% of cases.

After a median duration of first-line treatment of 3.7 months, 75% of patients obtained a response according to RECIST criteria, with significant reductions of the median size of the largest lesions (42 versus 25 mm, *P* < 0.001) and median number of liver metastases (4 versus 3, *P* < 0.001). Patients with bilobar disease and lesions in contact with major hepatic vessels decreased after conversion chemotherapy, as well (59% versus 49%, *P* < 0.0001, and 63% versus 59%, *P* = 0.04, respectively). At least one CRLM disappeared in 67% of cases. In addition, a significant reduction in CEA levels (*P* < 0.001) was reported. A higher number of patients underwent MRI before surgery as compared to baseline (49% versus 34%, *P* = 0.0001). Overall, at least one preoperative (baseline and/or pre-surgery) MRI scan was carried out in 61% of patients.

Most liver surgeries were carried out with one-stage procedures (91%), while concurrent ablation was used in 51 cases (19%) obtaining R0 and R1 resections in 200 (75%) and 68 (25%) patients, respectively (Supplementary Figure S1, available at https://doi.org/10.1016/j.esmoop.2024.102991). Post-operative chemotherapy was administered to 151 (56%) patients.

After a median follow-up of 92.3 months, 207 (77%) out of 268 patients experienced disease relapse. Among them, 96 (46%) patients had early recurrence within 6 months from liver surgery (Supplementary Figure S1, available at https://doi.org/10.1016/j.esmoop.2024.102991). In the overall population, unresected primary tumour at baseline (*P* = 0.009), a high number of baseline CRLMs (*P* = 0.07) and bilobar disease at baseline (*P* = 0.046) and pre-surgery (*P* = 0.08) were associated with early recurrence at univariate analyses. However, only primary tumour resection at baseline (OR 0.53, 95% CI 0.32-0.89, *P* = 0.02) retained statistical significance at the multivariate model (Table 2). Among patients with resected primary tumour at baseline, only pN+ was associated with early recurrence (OR 3.02, 95% CI 1.23-7.41, *P* = 0.02) (Supplementary Table S1, available at https://doi.org/10.1016/j.esmoop.2024.102991). Using the 12-month cut-off, 149 out of 207 relapsed patients (72%) experienced early recurrence. Although several variables were associated with early relapse at the univariate analyses, none of them retained statistical significance at the multivariate models (Table 2 and Supplementary Table S1, available at https://doi.org/10.1016/j.esmoop.2024.102991).

**Table 2:** Logistic regression model for disease relapse at 6 and 12 months after CRLM resection in the overall population

		Risk of relapse at 6 months after CRLM resection				Risk of relapse at 12 months after CRLM resection			
		Univariate analysis		Multivariate analysis		Univariate analysis		Multivariate analysis	
Factors	Nr.	OR and 95% CI	*P* value	OR and 95% CI	*P* value	OR and 95% CI	*P* value	OR and 95% CI	*P* value
**Age**									
≥65 years	112	0.87 (0.52-1.44)	0.58	—	—	0.71 (0.44-1.17)	0.18	—	—
<65 years	156	Reference				Reference			
**ECOG-PS**								—	—
1-2	34	1.83 (0.88-3.79)	0.11	—	—	1.29 (0.61-2.75)	0.50	—	—
0	220	Reference				Reference			
NA	14								
**Primary tumour resected at baseline**									
Yes	154	0.51 (0.31-0.85)	**0.009**	0.53 (0.32-0.89)	**0.02**	0.62 (0.37-1.02)	**0.06**	0.70 (0.41-1.19)	0.19
No	114	Reference				Reference			
**CRLM diagnosis**									
Synchronous	219	1.33 (0.68-2.59)	0.40	—	—	1.28 (0.69-2.40)	0.43	—	—
Metachronous	49	Reference				Reference			
**Primary tumour location**									
Left or rectum	194	0.82 (0.47-1.42)	0.48	—	—	0.89 (0.51-1.54)	0.66	—	—
Right	74	Reference				Reference			
Adjuvant chemotherapy									
Yes	19	1.05 (0.40-2.76)	0.93	—	—	1.15 (0.44-3.02)	0.78	—	—
No	259	Reference				Reference			
**Baseline CEA (continuous)**	222	1.00 (1.00-1.00)	0.34	—	—	1.00 (1.00-1.00)	0.50	—	—
NA	46			—	—				
**Baseline CEA (dichotomous)**									
≥10	95	1.48 (0.84-2.60)	0.17	—	—	1.39 (0.81-2.40)	0.23	—	—
<10	127	Reference				Reference			
NA	46								
**Pre-surgery CEA (continuous)**	176	1.00 (1.00-1.00)	0.59	—	—	1.00 (1.00-1.00)	0.68	—	—
NA	92				—			—	
**Pre-surgery CEA (dichotomous)**									
≥10	53	1.38 (0.72-2.66)	0.33	—	—	1.72 (0.85-3.51)	0.13	—	—
<10	123	Reference		—	—	Reference			
NA	92							—	—
**Baseline liver lobe involvement**									
Unilobar	111	0.59 (0.35-0.99)	**0.046**	0.68 (0.29-1.59)	0.37	0.44 (0.27-0.73)	**0.001**	0.50 (0.19-1.29)	0.15
Bilobar	157	Reference				Reference			
**Pre-surgery liver lobe involvement**									
Unilobar	131	0.64 (0.38-1.05)	**0.08**	0.99 (0.43-2.25)	0.98	0.54 (0.33-0.89)	**0.02**	1.03 (0.41-2.57)	0.96
Bilobar	137	Reference				Reference			
**Baseline Nr of liver segments involved**	268	1.05 (0.91-1.20)	0.51	—	—	1.24 (1.08-1.45)	**0.003**	0.95 (0.63-1.43)	0.81
**Baseline Nr of liver segments involved (dichotomous)**									
≥4	141	1.18 (0.71-1.94)	0.53	—	—	2.03 (1.24-3.34)	**0.005**	1.20 (0.41-3.20)	0.75
<4	127	Reference				Reference			
**Pre-surgery Nr of liver segments involved (continuous)**	268	1.01 (0.88-1.17)	0.84	—	—	1.09 (0.95-1.24)	0.24	—	—
**Pre-surgery Nr of liver segments involved (dichotomous)**									
≥4	104	1.05 (0.63-1.76)	0.84	—	—	1.44 (0.87-2.40)	0.16	—	—
<4	164	Reference				Reference			
**Baseline Nr of liver lesions (continuous)**	268	1.04 (0.99-1.09)	**0.07**	1.02 (0.98-1.07)	0.28	1.08 (1.01-1.15)	**0.02**	1.04 (0.96-1.14)	0.31
**Baseline Nr of liver lesions (dichotomous)**									
≥4	134	1.21 (0.74-2.00)	0.45	—	—	1.81 (1.11-2.98)	**0.02**	0.98 (0.41-2.30)	0.96
<4	134	Reference				Reference			
**Pre-surgery Nr of liver lesions (continuous)**	268	1.01 (0.97-1.06)	0.51	—	—	1.02 (0.98-1.07)	0.31	—	—
**Pre-surgery Nr of liver lesions (dichotomous)**									
≥4	111	1.09 (0.65-1.80)	0.75	—	—	1.41 (0.85-2.32)	0.18	—	—
<4	157	Reference				Reference			
**Nr of vanished CRLMs (continuous)**	268	1.08 (0.94-1.25)	0.27	—	—	1.23 (1.02-1.48)	**0.03**	1.14 (0.91-1.43)	0.24
**Vanished CRLMs (dichotomous)**									
Yes	181	0.92 (0.54-1.56)	0.75	—	—	1.51 (0.88-2.58)	0.13	—	—
No	87	Reference		—	—	Reference			
**Baseline max diameter of the largest liver lesion (continuous)**	268	1.00 (0.99-1.01)	0.90	—	—	1.00 (0.99-1.01)	0.12	—	—
**Pre-surgery max diameter of the largest liver lesion (continuous)**	268	1.01 (1.00-1.02)	0.13	—	—	1.01 (1.00-1.02)	**0.06**	1.06 (0.98-1.16)	0.25
**Baseline Nr of liver lesions in contact with vessels (continuous)**	268	0.98 (0.84-1.15)	0.79	—	—	1.11 (0.94-1.30)	0.21	—	—
**Baseline Nr of liver lesions in contact with vessels (dichotomous)**									
Yes	170	0.89 (0.52-1.47)	0.62	—	—	1.21 (0.73-2.00)	0.46	—	—
No	98	Reference		—	—	Reference		—	—
**Pre-surgery Nr of liver lesions in contact with vessels (continuous)**	268	1 (0.83-1.19)	0.98	—	—	1.02 (0.86-1.22)	0.77	—	—
**Pre-surgery Nr of liver lesions in contact with vessels (dichotomous)**									
Yes	158	0.90 (0.54-1.49)	0.68	—	—	1.07 (0.65-1.76)	0.78	—	—
No	110	Reference				Reference		—	—
**MRI with gadolinium-based contrast at baseline and/or before surgery**									
Yes	164	1.09 (0.65-1.82)	0.74	—	—	1.32 (0.59-2.99)	0.49	—	—
No	104	Reference				Reference			
***RAS* and *BRAF* mutational status**									
*RAS* MUT	140	1.50 (0.90-2.52)	0.12	—	—	0.90 (0.54-1.48)	0.67	—	—
*BRAF* MUT	6	1.09 (0.19-6.24)	0.91			3.08 (0.35-27.24)	0.31		
WT	118	Reference				Reference			
NA	4								
**Objective response to chemotherapy**									
Yes	202	1.16 (0.64-2.08)	0.63	—	—	0.82 (0.46-1.46)	0.50	—	—
No	66	Reference		—		Reference			
**Chemotherapy regimen**									
Triplet	112	0.87 (0.52-1.44)	0.58	—	—	1.19 (0.72-1.96)	0.49	—	—
Doublets	156	Reference				Reference			
**Biologic agent administered**									
Anti-EGFR	68	0.72 (0.28-1.85)	0.49	—	—	1.64 (0.64-4.20)	0.30	—	—
Anti-VEGF	175	0.87 (0.37-2.04)	0.74			1.08 (0.46-2.50)	0.87		
None	25	Reference				Reference			
**Duration of chemotherapy before surgery**									
≥3.7 months	134	0.94 (0.57-1.54)	0.80	—	—	0.67 (0.41-1.09)	0.11	—	—
<3.7 months	134	Reference		—	—				
**Scheduled liver surgery**									
One step	244	0.52 (0.23-1.22)	0.16			0.89 (0.38-2.12)	0.80	—	—
Two steps	24	Reference		—	—	Reference		—	—

*P*-value < 0.10 in univariate and < 0.05 in multivariate analyses are highlighted in bold.

CEA, carcinoembryonic antigen; CI, confidence interval; CRLM, colorectal liver metastasis; ECOG-PS, Eastern Cooperative Oncology Group performance status; EGFR, epidermal growth factor receptor; MRI, magnetic resonance imaging; MUT, mutant; NA, not available; Nr, number; OR, odds ratio; VEGF, vascular endothelial growth factor; WT, wild-type.

In the overall study population, median DFS and OS were 9.7 months (95% CI 8.06-11.55 months) and 49.7 months (95% CI 40.49-64.57 months), respectively (Supplementary Figure S2, available at https://doi.org/10.1016/j.esmoop.2024.102991). At multivariate analysis, only resection of the primary tumour at baseline (HR 0.54, 95% CI 0.37-0.78, *P* = 0.001) was independently associated with DFS, while ECOG-PS (HR 2.14, 95% CI 1.22-3.77, *P* = 0.008) and duration of preoperative chemotherapy (HR 0.60, 95% CI 0.37-0.97, *P* = 0.04) had a statistically significant effect in terms of OS (Table 3). Among patients with resected primary tumour at baseline, a pathological N+ stage (HR 1.72, 95% CI 1.09-2.71, *P* = 0.02) and *BRAF* mutation (HR 3.50, 95% CI 1.17-10.48, *P* = 0.03) had a statistically significant effect in terms of DFS (Table 3), while *BRAF* mutation (HR 4.88, 95% CI 1.42-16.78, *P* = 0.01) and ECOG-PS > 0 (HR 2.89, 95% CI 1.63-5.15, *P* = 0.0003) affected OS at multivariate analysis (Supplementary Table S2, available at https://doi.org/10.1016/j.esmoop.2024.102991).

**Table 3:** Cox regression model for DFS and OS after CRLM resection in the overall population

		Disease-free survival				Overall survival			
		Univariate analysis		Multivariate analysis		Univariate analysis		Multivariate analysis	
Factors	Nr.	HR and 95% CI	*P* value	HR and 95% CI	*P* value	HR and 95% CI	*P* value	HR and 95% CI	*P* value
**Age**									
≥65 years	112	0.92 (0.70-1.21)	0.92	—	—	1.27 (0.89-1.81)	0.20	—	—
<65 years	156	Reference		—	—	Reference		—	—
**ECOG-PS**									
1-2	34	1.29 (0.87-1.93)	0.21	—	—	2.64 (1.58-4.40)	**<0.0001**	2.14 (1.21-3.77)	**0.008**
0	220	Reference				Reference			
NA	14								
**Primary tumour resected at baseline**									
Yes	154	0.66 (0.50-0.87)	**0.0035**	0.54 (0.37-0.78)	**0.001**	0.78 (0.54-1.12)	0.17	—	—
No	114	Reference				Reference		—	
**Adjuvant chemotherapy**									
Yes	19	1.14 (0.67-1.92)	0.64	—	—	1.07 (0.50-2.30)	0.86	—	—
No	258	Reference		—	—	Reference		—	—
**CRLM diagnosis**									
Synchronous	219	1.45 (0.99-2.13)	**0.056**	0.92 (0.54-1.59)	0.78	1.30 (0.79-2.14)	0.31	—	—
Metachronous	49	Reference				Reference		—	—
**Primary tumour location**					—		0.34		
Left or rectum	194	1.18 (0.86-1.62)	0.30	—	—	0.83 (0.55-1.23)		—	—
Right	74	Reference		—		Reference		—	—
**Baseline CEA (continuous)**	222	1 (1.00 -1.01)	0.31	—	—	1 (1.00 -1.01)	0.80	—	—
**NA**	46			—	—			—	—
**Baseline CEA (dichotomous)**									
≥10	95	1.27 (0.93-1.73)	0.13	—	—	1.27 (0.93-1.73)	0.13	—	—
<10	127	Reference				Reference			
NA	46			—	—			—	—
**Pre-surgery CEA (continuous)**	176	1 (0.99-1.00)	0.84	—	—	1 (0.99-1.00)	0.84	—	—
NA	92	Reference		—	—	Reference			
**Pre-surgery CEA (dichotomous)**									
≥10	53	1.50 (1.05-2.14)	**0.03**	1.17 (0.76-1.81)	0.47	1.41 (0.95-2.12)	**0.09**	1.46 (0.90-2.39)	0.13
<10	123	Reference				Reference			
NA	92								
**Baseline liver lobe involvement**									
Unilobar	111	0.67 (0.51-0.90)	**0.006**	0.56 (0.31-1.00)	0.051	0.71 (0.49-1.02)	**0.07**	0.62 (0.37-1.03)	0.07
Bilobar	157	Reference				Reference			
**Pre-surgery liver lobe involvement**									
Unilobar	131	0.75 (0.57-0.99)	**0.04**	1.30 (0.75-2.27)	0.35	1.06 (0.74-1.52)	0.73	—	—
Bilobar	137	Reference				Reference		—	—
**Baseline Nr of liver segments involved**	268	1.10 (1.02-1.19)	**0.009**	0.84 (0.67-1.05)	0.12	0.96 (0.86-1.06)	0.40	—	—
**Baseline Nr of liver segments involved (dichotomous)**									
≥4	141	1.40 (1.06-1.85)	**0.02**	1.52 (0.76-3.05)	0.23	0.99 (0.69-1.42)	0.97	—	—
<4	127	Reference				Reference		—	—
**Pre-surgery Nr of liver segments involved (continuous)**	268	1.06 (0.98-1.14)	0.12	—	—	1.03 (0.93-1.14)	0.56	—	—
**Pre-surgery Nr of liver segments involved (dichotomous)**									
≥4	104	1.26 (0.96-1.67)	**0.09**	0.91 (0.48-1.72)	0.77	0.83 (0.57-1.22)	0.34	—	—
<4	164	Reference				Reference		—	—
**Baseline Nr of liver lesions (continuous)**	268	1.03 (1.02-1.05)	**0.0002**	1.03 (1.00-1.07)	0.08	1.02 (1.00-1.05)	**0.047**	1.00 (0.97-1.03)	0.93
**Baseline Nr of liver lesions (dichotomous)**									
≥4	134	1.49 (1.13-1.97)	**0.005**	0.97 (0.45-2.09)	0.94	1.26 (0.88-1.80)	0.20	—	—
<4	134	Reference				Reference		—	—
**Pre-surgery Nr of liver lesions (continuous)**	268	0.99 (0.95-1.03)	0.50	—	—	1.01 (0.98-1.04)	0.44	—	—
**Pre-surgery Nr of liver lesions (dichotomous)**									
≥4	111	1.02 (1.00-1.04)	**0.02**	1.13 (0.53-2.43)	0.75	1.04 (0.73-1.50)	0.81	—	—
<4	157	Reference				Reference		—	—
**Nr of vanished CRLMs (continuous)**	268	1.08 (1.00-1.17)	**0.06**	0.94 (0.80-1.11)	0.49	1.10 (1.02-1.19)	**0.02**	0.97 (0.83-1.14)	0.72
**Vanished CRLMs (dichotomous)**									
Yes	181	1.09 (0.82-1.46)	0.54	—	—	1.32 (0.91-1.92)	0.15	—	—
No	87	Reference		—	—	Reference		—	—
**Baseline max diameter of the largest liver lesion (continuous)**	268	1 (0.99-1.01)	0.16	—	—	1 (1.00 -1.01)	0.51	—	—
**Pre-surgery max diameter of the largest liver lesion (continuous)**	268	1.00 (1.00-1.01)	**0.04**	1.01 (1.00-1.02)	0.054	1.00 (1.00-1.01)	0.15	—	—
**Baseline Nr of liver lesions in contact with vessels (continuous)**	268	1.08 (0.99-1.17)	**0.06**	0.98 (0.83-1.16)	0.84	1.04 (0.93-1.17)	0.47	—	—
**Baseline Nr of liver lesions in contact with vessels (dichotomous)**									
Yes	170	1.22 (0.91-1.62)	0.18	—	—	1.17 (0.80-1.71)	0.41	—	—
No	98	Reference		—	—	Reference		—	—
**Pre-surgery Nr of liver lesions incontact with vessels (continuous)**	268	1.07 (0.98-1.18)	0.14	—	—	0.98 (0.85-1.13)	0.78	—	—
**Pre-surgery Nr of liver lesions in contact with vessels (dichotomous)**									
Yes	158	1.15 (0.87-1.53)	0.31	—	—	1.09 (0.76-1.57)	0.64	—	—
No	110	Reference		—	—	Reference		—	—
**MRI with gadolinium-based contrast at baseline and/or before surgery**									
Yes	164	1.00 (0.76-1.32)	0.99	—	—	0.91 (0.64-1.30)	0.61	—	—
No	104	Reference		—	—	Reference		—	—
***RAS* and *BRAF* mutational status**									
*RAS* MUT	140	1.09 (0.82-1.44)	0.54	—	—	1.38 (0.96-1.99)	**0.09**	1.53 (0.96-2.46)	0.08
*BRAF* MUT	6	1.62 (0.65-3.98)	0.30			1.97 (0.61-6.35)	0.26	1.73 (0.41-7.32)	0.46
WT	118	Reference				Reference			
NA	4			—	—				
**Objective response to chemotherapy**									
Yes	202	0.90 (0.66-1.23)	0.50	—	—	0.83 (0.56-1.25)	0.38	—	—
No	66	Reference				Reference			
**Chemotherapy regimen**									
Triplet	112	1.09 (0.82-1.44)	0.55	—	—	1.31 (0.91-1.87)	0.14	—	—
Doublets	156	Reference		—	—	Reference		—	—
**Biologic agent administered**									
Anti-EGFR	68	1.57 (0.91-2.71)	0.11	—	—	0.92 (0.48-1.96)	0.92	—	—
Anti-VEGF	175	1.32 (0.80-2.19)	0.28			0.69 (0.61-2.13)	0.69		
None	25	Reference		—	—	Reference		—	—
**Duration of chemotherapy before surgery**									
≥3.7 months	134	0.80 (0.61-1.05)	**0.102**	0.95 (0.66-1.36)	0.95	0.65 (0.45-0.93)	**0.02**	0.60 (0.37-0.97)	**0.04**
<3.7									
months	134	Reference				Reference			
**Scheduled surgery**									
One step	244	0.87 (0.55-1.39)	0.57	—	—	0.95 (0.50-1.83)	0.89	—	—
Two steps	24	Reference		—	—	Reference		—	—

*P*-value < 0.10 in univariate and < 0.05 in multivariate analyses are highlighted in bold.

CEA, carcinoembryonic antigen; CI, confidence interval; CRLM, colorectal liver metastasis; DFS, disease-free survival; ECOG-PS, Eastern Cooperative Oncology Group performance status; EGFR, epidermal growth factor receptor; HR, hazard ratio; MRI, magnetic resonance imaging; MUT, mutant; NA, not available; Nr, number; OR, odds ratio; OS, overall survival; VEGF, vascular endothelial growth factor; WT, wild-type.

**Figure 1:**
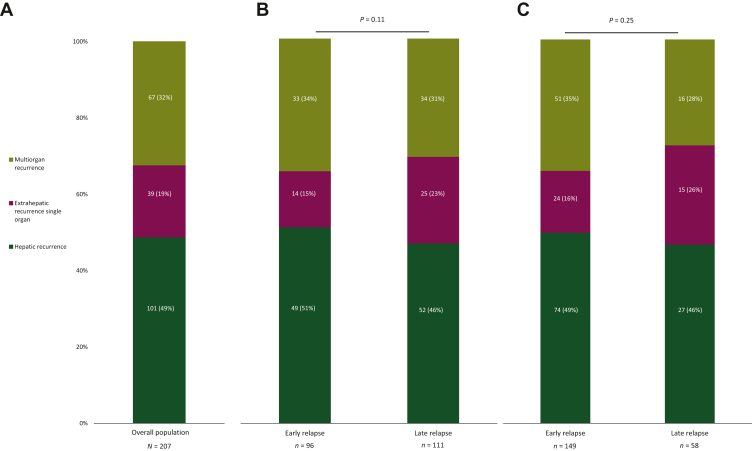
**Patterns of disease recurrence**. In the overall population (A) and according to timing of disease relapse, with cut-offs for early recurrence of 6 months (B) and 12 months (C).

**Figure 2:**
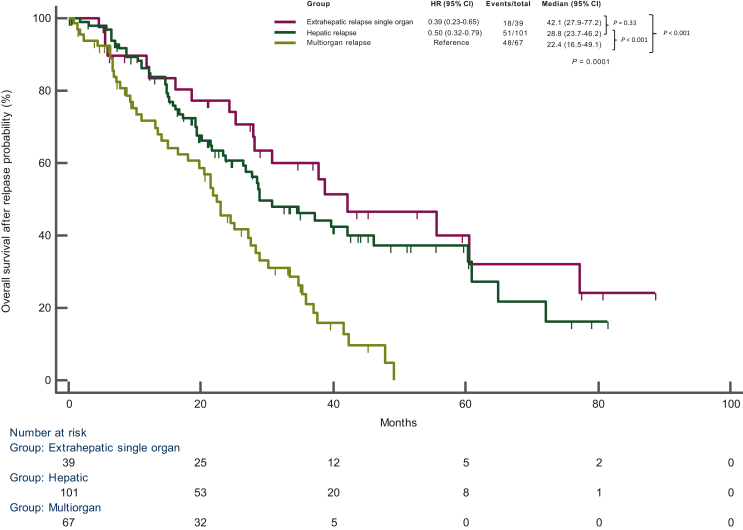
**Overall survival from disease relapse according to patterns of disease recurrence**. CI, confidence interval; HR, hazard ratio.

As expected, among 207 patients experiencing disease recurrence, patients with late recurrence showed a longer post-relapse OS compared with the early recurrence group using both 6 and 12 months as cut-off (*P* < 0.0001) (Supplementary Figure S3, available at https://doi.org/10.1016/j.esmoop.2024.102991). Hepatic-only, extrahepatic single-organ and multiorgan relapse occurred in 101 (49%), 39 (19%) and 67 (32%) patients, respectively (Figure 1 A). A shorter post-relapse OS was observed in patients with multiorgan recurrence with respect to other groups (*P* < 0.001) (Figure 2).

No difference was observed between late and early recurrence in terms of site of relapse using both 6- (*P* = 0.11) and 12-month (*P* = 0.25) cut-offs. (Figure 1 B and C).

Using a cut-off of 6 months, a longer post-relapse OS was reported in patients with late hepatic-only compared to early hepatic-only recurrence (*P* = 0.002) and in subjects with late multiorgan relapse with respect to early multiorgan relapse (*P* = 0.04), while no difference was observed between late extrahepatic single-organ and early extrahepatic single-organ recurrence (*P* = 0.14) (Supplementary Figure S4 A, available at https://doi.org/10.1016/j.esmoop.2024.102991). When a cut-off of 12 months was used, a longer post-relapse OS was reported in patients with late hepatic-only compared to early hepatic-only recurrence (*P* = 0.046) and in subjects with late extrahepatic single-organ with respect to early extrahepatic single-organ (*P* = 0.03), while no difference was observed between late multiorgan and early multiorgan recurrence (*P* = 0.40) (Supplementary Figure S4, panel B, available at https://doi.org/10.1016/j.esmoop.2024.102991).

After relapse, at least one subsequent locoregional treatment (LRT) with curative intent was carried out in 92 patients (44%), including 22 subjects (11%) receiving more than one subsequent LRT. A statistically significant higher percentage of subsequent LRTs were carried out among patients experiencing late recurrence using a 6-month cut-off, (51% versus 36%, *P* = 0.03) but not when a 12-month cut-off was adopted (50% versus 42%, *P* = 0.32) (Supplementary Figure S5, panel A-C, available at https://doi.org/10.1016/j.esmoop.2024.102991). Liver LRTs accounted for 76% of cases (54 liver surgery and 15 non-surgical LRTs), followed by 16% of lung LRTs (13 surgery and 2 radiotherapy), 4% of other single-organ LRTs and 4% of other multiorgan LRTs. Using a 6-month cut-off, early relapses were associated with a statistically significant higher frequency of non-surgical liver LRTs (31% versus 7%, *P* = 0.02) and a lower frequency of lung surgery (6% versus 19%, *P* = 0.003) with a numerically lower percentage of liver resections (48% versus 64%, *P* = 0.12), as compared to late relapses. Results with the 12-month cut-off are described in Supplementary Figure S5 D-F, available at https://doi.org/10.1016/j.esmoop.2024.102991. The delivery of one or more LRTs after disease progression was associated with longer post-relapse OS (60.3 versus 19.1 months, HR 0.19, 95% CI 0.13-0.29, *P* < 0.0001) (Supplementary Figure S6, available at https://doi.org/10.1016/j.esmoop.2024.102991). Furthermore, after stratifying subjects according to timing of disease relapse, LRTs after relapse were associated with longer post-resection OS both among early and late recurring patients (*P* < 0.001) (Supplementary Figure S7 A and B, available at https://doi.org/10.1016/j.esmoop.2024.102991).

## Discussion

Although CRLM resection is a unique chance of cure for mCRC patients, or at least it significantly enhances survival for a group of mCRC patients, roughly half of the patients relapse within 6-12 months from liver metastasectomy, thus raising concerns about the usefulness of surgery in those cases, also considering the related risk of perioperative mortality and severe morbidity.^6^^,^^11^^,^^24^^,^^25^ Moreover, it should be noticed that maintenance with a fluoropyrimidine plus the targeted agent initially used is a safe option, with a relatively low burden of adverse events, able to prolong the time to disease progression, with a median duration of clinical benefit of 8 months following 4-6 months of upfront combination therapy and no demonstrated survival benefit. However, our current ability to select patients with a maximized benefit from liver surgery is quite poor.

Therefore, with the aim of estimating the risk of early recurrence and thus supporting clinicians and patients for the choice of the most appropriate approach in each individual patient amenable of liver resection after upfront chemotherapy, we retrospectively assessed the association of baseline and pre-surgical clinical, radiological and molecular factors with early relapse in a cohort of initially unresectable patients with liver-limited CRLMs who underwent secondary surgery at three Italian Hospitals with high-volume centres for liver surgery.

In our study, none of the investigated factors was found to be an independent predictor of 6-month recurrence, with the exception of unresected primary tumour at baseline and, among patients with resected primary tumour, pN+ stage. Even using a 12-month threshold, we failed to show any baseline and pre-surgical factor that independently correlated with the risk of early relapse. Accordingly, the multivariate Cox regression analysis did not identify any baseline or pre-surgical marker associated with improved survival to be included in a prognostic model. Only the primary tumour resection at baseline was associated with longer DFS in the overall population and *BRAF* mutation, and pN+ stage predicted shorter DFS among patients with resected primary tumour. In spite of apparently disappointing results, our findings actually confirm and expand a recent *post hoc* analysis of the Dutch CAIRO5 trial, where patients with initially unresectable CRLMs underwent bimonthly re-assessments for resectability by a central panel of experts in liver surgery during the administration of the most active first-line regimen according to international guidelines.^11^ Notably, despite an enormous effort for multidimensional centralized revision in a randomized trial, only the number of liver metastases before local treatment was able to predict recurrence within 6 months,^11^ thus not allowing to build a predictive model of early relapse potentially useful for clinical decision making in patients candidate to liver surgery after upfront chemotherapy. Of note, although the median number of baseline liver lesions was 12 and 4 in the CAIRO5 study and in our cohort, respectively, according to the different definitions of unresectability (unresectable at baseline if an R0 resection could not be achieved in a single procedure by surgical resection versus technical/surgical and oncological/prognostic criteria), the similar results of our retrospective real-life cohort with those from the prospective investigational CAIRO5 study with comparable population size (*n* = 268 and *n* = 240, respectively) and frequencies of early disease relapse (36% and 43%, respectively) strengthens the reliability of our findings and the weight of oncological/prognostic factors on the probability of early relapse.^11^ Therefore, even if a median number of four liver metastases may appear too small to define our cohort as ‘initially unresectable’, several additional technical/surgical and oncological/prognostic criteria contribute to the definition of resectability.^1^^,^^13^ Indeed, most of the patients in our cohort had synchronous metastases, bilobar liver involvement with more than four hepatic segments affected, a median size of the largest lesion >40 mm, metastases in contact with at least one major hepatic vessel, *RAS* mutation and pathologic lymph nodes when primary tumour was resected. Unfortunately, we cannot state which specific criteria (technical, oncological or both) actually led to exclude the initial resectability in each individual case, and therefore we are not able to distinguish the impact of oncological/prognostic criteria versus technical/surgical ones in our population.

Overall, our work remarks the unreliability of the current clinical, radiological and molecular features—evaluated either before or after chemotherapy—to spare futile surgery in those patients at high risk of disease relapse after conversion chemotherapy. Notably, in our study, half of the patients with early recurrence reported a post-relapse OS longer than 27 months, thus suggesting that some patients with early recurrence still achieve benefit from liver surgery, although the potential survival benefit of metastasectomy in those patients experiencing early recurrence could not be adequately evaluated, due to the lack of a comparator arm consisting of patients not receiving secondary resection, which would be probably unethical, considering the high benefit of liver surgery. In addition, patients with early relapse, especially those experiencing single-organ recurrence, may benefit from further locoregional approaches, that were still feasible in more than one-third of the patients experiencing disease relapse within 6 months from liver surgery. Overall, survival was prolonged by LRT administered after first recurrence irrespective of the timing of relapse, thus leading to consider this approach whenever feasible.

We acknowledge some clear limitations of our study, including the retrospective design, the long timeframe of patients’ inclusion, the heterogeneity of administered first-line treatments, though mostly including the same regimens administered in the CAIRO5 trial, and the availability of baseline and pre-surgical liver MRI for roughly one-third and a half of included patients, respectively. Of note, the number of variables considered in our prognostic analysis implies a risk of false-positive results intrinsic in multiple tests, and—even more importantly—the absence of statistical significance for the vast majority of the variables tested should be cautiously interpreted considering the risk of false-negative results. If we had applied the widely adopted rule-of-thumb of a minimum number of events (i.e. 10) for candidate predictor parameter, we would have needed a higher sample size.^26^ However, more flexible approaches have been proposed for clinical prediction models,^27^ and our sample size (in terms of number of patients and events) can be considered adequate to estimate with enough accuracy the main outcome (the probability of early relapse). Our study also has strengths, including the revision of imaging by radiologists with liver imaging expertise and the inclusion of patients assessed in high-volume liver surgery institutions.

Considering the inaccuracy of investigated predictors for the early relapse, the most appropriate treatment option remains to offer surgery as soon as the disease becomes resectable. In addition, we endorse the adoption of novel approaches to estimate the risk of early disease relapse in the multidimensional assessment of patients with initially unresectable CRLMs. To this purpose, the longitudinal assessment of circulating tumour DNA (ctDNA) could be an ideal, non-invasive tool to track the disease load before and after conversion chemotherapy able to predict the risk of early recurrence. Similarly, the dynamic evaluation of radiomic features and the semi-quantitative analysis of apparent diffusion coefficient (ADC) on the diffusion-weighted liver MRI sequences^28^ could help obtain useful information about the probability of early relapse.^29^, ^30^, ^31^, ^32^ The accuracy and reproducibility of ctDNA profiling, extensively investigated in the post-operative setting of resected localized and metastatic colorectal cancer, radiomic signatures and ADC evaluation, deserve prospective investigation in the pre-operatory setting of patients with initially unresectable CRLMs.^33^, ^34^, ^35^, ^36^

Based on our findings, the secondary resection of CRLMs should be never denied to initially unresectable patients that become resectable after upfront chemotherapy.
