# Supplementary material for: Predicting early recurrence after resection of initially unresectable colorectal liver metastases: the role of baseline and pre-surgery clinical, radiological and molecular factors in a real-life multicentre experience
Source: ESMO Open. 2024 Apr 16;9(4):102991. doi: 10.1016/j.esmoop.2024.102991 (PMC11027482; doi:10.1016/j.esmoop.2024.102991)
Supplement: Supplemental Table 1 [file mmc1.docx]

| Supplementary Table 1  Logistic regression model for disease relapse at 6 and 12 months after CRLM resection in baseline resected primary tumour population | | | | | | | | | |
| --- | --- | --- | --- | --- | --- | --- | --- | --- | --- |
|  | | Risk of relapse at 6 months after CRLM resection | | | | Risk of relapse at 12 months after CRLM resection | | | |
|  |  | Univariate analysis | | Multivariate analysis | | Univariate analysis | | Multivariate analysis | |
| Factors | Nr. | OR and 95% CI | p value | OR and 95% CI | p value | OR and 95% CI | p value | OR and 95% CI | p value |
| Age  ≥ 65 years  < 65 years | 68  86 | 0.90 (0.44-1.81)  Reference | 0.76 | - | **-** | 0.69 (0.36-1.30)  Reference | 0.25 | - | - |
| ECOG PS  1-2  0  NA | 131  20  3 | 1.76 (0.67-4.66)  Reference | 0.26 | - | **-** | 1.22 (0.47-3.21)  Reference | 0.67 | - | - |
| CRLM diagnosis  Synchronoous  Metachrnous | 109  45 | 1.19 (0.55-2.60)  Reference | 0.65 | **-** | - | 1.26 (0.63-2.53)  Reference | 0.51 | **-** | - |
| Primary tumour location  Left or rectum  Right | 112  42 | 0.77 (0.36-1.64)  Reference | 0.49 | - | - | 1.02 (0.50-2.09)  Reference | 0.95 | - | - |
| pT stage  pT4  pT1-T3 | 31  123 | 0.98 (0.41-2.33)  Reference | 0.96 | - | - | 1.34 (0.60-3.00)  Reference | 0.47 | - | - |
| pN stage  pN1-2  pN0 | 108  46 | 3.02 (1.23-7.41)  Reference | **0.02** | - | - | 1.96 (0.98-3.95)  Reference | **0.06** | 1.64 (0.76-3.50) | 0.21 |
| Adjuvant chemotherapy  Yes  No | 19  135 | 1.49 (0.54-4.07)  Reference | 0.44 | **-** | - | 1.45 (0.54-3.93)  Reference | 0.46 | **-** | - |
| Baseline CEA (continuous)  NA | 132  22 | 1.00 (1.00-1.00) | 0.35 | **-** | - | 1.00 (1.00-1.00) | 0.48 | **-** | - |
| Baseline CEA (dichotomous)  ≥ 10  < 10  NA | 63  69  22 | 1.42 (0.66-3.05)  Reference | 0.37 | - | - | 1.31 (0.66-2.60)  Reference | 0.45 | - | - |
| Pre-surgery CEA (continuous)  NA | 98  56 | 1.00 (1.00-1.00) | 0.51 | - | - | 1.00 (1.00-1.00) | 0.67 | - | - |
| Pre-surgery CEA (dichotomous)  ≥ 10  < 10  NA | 27  71  56 | 1.75 (0.70-4.42)  Reference | 0.24 | - | - | 1.48 (0.59-3.67)  Reference | 0.40 | - | - |
| Baseline Liver lobe involvement  Unilobar  Bilobar | 68  86 | 0.61 (0.29-1.24)  Reference | 0.17 | - | - | 0.40 (0.21-0.77)  Reference | **0.006** | 0.55 (0.17-1.75) | 0.31 |
| Pre-surgery Liver lobe involvement  Unilobar  Bilobar | 82  72 | 0.69 (0.34-1.38)  Reference | 0.29 | - | - | 0.57 (0.30-1.09)  Reference | **0.09** | 1.04 (0.33-3.34) | 0.95 |
| Baseline Nr of liver segments involved | 154 | 0.98 (0.80-1.20) | 0.87 | - | - | 1.24 (1.02-1.51) | **0.03** | 0.98 (0.68-1.42) | 0.92 |
| Baseline Nr of liver segments involved (dichotomous)  ≥ 4  < 4 | 71  83 | 1.03 (0.52-2.08)  Reference | 0.93 | - | - | 2.08 (1.08-3.98)  Reference | **0.03** | 1.58 (0.45-5.52) | 0.48 |
| Pre-surgery Nr of liver segments involved (continuous) | 154 | 0.93 (0.76-1.14) | 0.50 | - | - | 1.05 (0.88-1.27) | 0.58 | - | - |
| Pre-surgery Nr of liver segments involved (dichotomous)  ≥ 4  < 4 | 50  104 | 0.92 (0.43-1.93)  Reference | 0.82 | - | - | 1.18 (0.60-2.34)  Reference | 0.63 | - | - |
| Baseline Nr of liver lesions (continuous) | 154 | 1.01 (0.96-1.07) | 0.53 | - | - | 1.06 (0.98-1.16) | 0.16 | - | - |
| Baseline Nr of liver lesions (dichotomous)  ≥ 4  < 4 | 70  84 | 1.21 (0.61-2.44)  Reference | 0.58 | **-** | - | 1.98 (1.03-3.79)  Reference | **0.04** | 0.84 (0.30-2.39) | 0.75 |
| Pre-surgery Nr of liver lesions (continuous) | 154 | 1.00 (0.95-1.05) | 0.93 | - | - | 1.01 (0.96-1.06) | 0.76 | - | - |
| Pre-surgery Nr of liver lesions (dichotomous)  ≥ 4  < 4 | 56  98 | 1.09 (0.53-2.23)  Reference | 0.82 | - | - | 1.42 (0.73-2.77)  Reference | 0.30 | - | - |
| Nr of vanished CRLMs (continuous) | 154 | 1.06 (0.83-1.34) | 0.66 | - | - | 1.34 (1.02-1.76) | **0.04** | 1.22 (0.86-1.74) | 0.26 |
| Vanished CRLMs (Dichotomous)  Yes  No | 48  106 | 0.86 (0.40-1.84)  Reference | 0.70 | - | - | 1.36 (0.68-2.72)  Reference | 0.38 | - | - |
| Baseline Max diameter of the largest liver lesion(continuous) | 154 | 1.00 (0.98-1.01) | 0.46 | **-** | - | 1.01 (0.99-1.02) | 0.38 | **-** | - |
| Pre-surgery Max diameter of the largest liver lesion(continuous) | 154 | 1.01 (0.99-1.02) | 0.41 | - | - | 1.01 (0.99-1.02) | 0.23 | - | - |
| Baseline Nr of liver lesions in contact with vessels (continuous) | 154 | 0.96 (0.76-1.21) | 0.73 | - | - | 1.11 (0.89-1.37) | 0.35 | - | - |
| Baseline Nr of liver lesions in contact with vessels (dichotomous)  Yes  No | 93  61 | 0.66 (0.33-1.34)  Reference | 0.25 | - | - | 1.20 (0.63-2.30)  Reference | 0.58 | - | - |
| Pre-surgery Nr of liver lesions in contact with vessels (continuous) | 154 | 0.89 (0.67-1.18) | 0.42 | **-** | - | 0.94 (0.75-1.18) | 0.58 | **-** | - |
| Pre-surgery Nr of liver lesions in contact with vessels (dichotomous)  Yes  No | 87  67 | 0.74 (0.37-1.48)  Reference | 0.39 | - | - | 1.00 (0.53-1.89)  Reference | 0.99 | - | - |
| MRI with gadolinium-based contrast at baseline and/or before surgery  Yes  No | 101  53 | 1.66 (0.77-3.57)  Reference | 0.20 | - | - | 1.30 (0.67-2.53)  Reference | 0.44 | - | - |
| *RAS* and *BRAF* mutational status  *RAS* MUT  *BRAF* MUT  WT  NA | 76  4  72  2 | 1.28 (0.63-2.63)  2.79 (0.37-21.22)  Reference | 0.49  0.32 | - | - | 0.61 (0.31-1.16)  4.77·10^8^*  Reference | 0.13  1.00 | - | - |
| Objective response to chemotherapy  Yes  No | 108  46 | 1.46 (0.66-3.22)  Reference | 0.35 | - | - | 0.92 (0.46-1.86)  Reference | 0.93 | - | - |
| Chemotherapy regimen  Triplet  Doublets | 58  96 | 0.77 (0.37-1.59)  Reference | 0.48 | - | - | 1.12 (0.58-2.15) | 0.74 | - | - |
| Biologic agent administered  Anti-EGFR  Anti-VEGF  None | 41  98  15 | 0.89 (0.23-3.42)  1.27 (0.38-4.31)  Reference | 0.86  0.70 | - | - | 2.20 (0.66-7.34)  1.24 (0.42-3.69) | 0.20  0.70 | - | - |
| Duration of chemotherapy before surgery  ≥ 3.7 months  < 3.7 months | 76  78 | 0.67 (0.33-1.34)  Reference | **0.26** | - | **-** | 0.43 (0.22-0.82) | **0.01** | 0.52 (0.26-1.04) | 0.07 |
| Scheduled liver surgery  One step  Two steps | 149  5 | 0.61 (0.10-3.77)  Reference | 0.59 | - | - | 0.82 (0.13-5.03)  Reference | 0.80 | - | - |

**Legend**

CI: confidence interval; CRLM: colorectal liver metastasis; MRI: magnetic resonance imaging; MUT: mutant; NA: not available; Nr: number; OR: odds ratio; WT: wild-type.

*Confidence interval could not be computed
