# Supplementary material for: Predicting early recurrence after resection of initially unresectable colorectal liver metastases: the role of baseline and pre-surgery clinical, radiological and molecular factors in a real-life multicentre experience
Source: ESMO Open. 2024 Apr 16;9(4):102991. doi: 10.1016/j.esmoop.2024.102991 (PMC11027482; doi:10.1016/j.esmoop.2024.102991)
Supplement: Supplemental Table 2 [file mmc2.docx]

| Supplementary Table 2  Cox regression model for DFS and OS after CRLM resection in baseline resected primary tumour population | | | | | | | | | |
| --- | --- | --- | --- | --- | --- | --- | --- | --- | --- |
|  | | Disease-free Survival | | | | Overall survival | | | |
|  |  | Univariate analysis | | Multivariate analysis | | Univariate analysis | | Multivariate analysis | |
| Factors | Nr. | HR and 95% CI | p value | HR and 95% CI | p value | HR and 95% CI | p value | HR and 95% CI | p value |
| Age at the diagnosis of CRLMs  ≥ 65 years  < 65 years | 68  86 | 0.83 (0.57-1.21)  Reference | 0.34 | - | **-** | 1.31 (0.81-2.11)  Reference | 0.27 | - | - |
| ECOG PS  1-2  0  NA | 131  20  3 | 1.28 (0.75-2.17)  Reference | 0.37 | - | **-** | 2.98 (1.70-5.19)  Reference | **0.0001** | 2.89 (1.63-5.15) | **0.0003** |
| CRLM diagnosis  Synchronoous  Metachrnous | 109  45 | 1.41 (0.91-2.17)  Reference | 0.12 | - | **-** | 1.29 (0.74-2.26)  Reference | 0.37 | - | - |
| Primary tumour location  Left or rectum  Right | 112  42 | 1.50 (0.95-2.36)  Reference | **0.08** | 1.22 (0.77-1.93) | 0.39 | 0.91 (0.53-1.56)  Reference | 0.73 | - | - |
| pT stage  pT4  pT1-T3 | 31  123 | 1.26 (0.79-1.98)  Reference | 0.33 | - | - | 1.48 (0.83-2.62)  Reference | 0.19 | - | - |
| pN stage  pN1-2  pN0 | 108  46 | 1.84 (1.19-2.84)  Reference | **0.006** | 1.72 (1.09-2.71) | **0.02** | 1.94 (1.10-3.42)  Reference | **0.02** | 1.36 (0. 75-2.45) | 0.31 |
| Adjuvant chemotherapy  Yes  No | 19  135 | 1.39 (0.81-2.39)  Reference | 0.24 | **-** | - | 1.22 (0.56-2.68)  Reference | 0.62 | - | - |
| Baseline CEA (continuous)  NA | 132  22 | 1.00 (1.00-1.00) | 0.41 | - | - | 1.00 (1.00-1.00) | 0.42 | - | - |
| Baseline CEA (dichotomous)  ≥ 10  < 10  NA | 63  69  22 | 1.38 (0.92-2.08)  Reference | 0.12 | - | - | 1.32 (0.83-2.08)  Reference | 0.24 | - | - |
| Pre-surgery CEA (continuous)  NA | 98  56 | 1.00 (1.00-1.00) | 0.85 | - | - | 1.27 (0.93-1.73) | 0.13 | - | - |
| Pre-surgery CEA (dichotomous)  ≥ 10  < 10  NA | 27  71  56 | 1.53 (0.92-2.57)  Reference | 0.11 | - | - | 1.25 (0.67-2.34)  Reference | 0.48 | - | - |
| Baseline Liver lobe involvement  Unilobar  Bilobar | 68  86 | 0.68 (0.47-0.99)  Reference | **0.046** | 0.96 (0.59-1.57) | 0.88 | 0.63 (0.38-1.02)  Reference | **0.07** | 0.74 (0.42-1.29) | 0.28 |
| Pre-surgery Liver lobe involvement  Unilobar  Bilobar | 82  72 | 0.80 (0.55-1.15)  Reference | 0.23 | - | **-** | 1.10 (0.68-1.78)  Reference | 0.69 | - | - |
| Baseline Nr of segments involved (continuous) | 154 | 1.12 (1.01-1.25)  Reference | **0.03** | 0.93 (0.75-1.16) | 0.50 | 1.00 (0.87-1.14)  Reference | 0.96 | - | - |
| Baseline Nr of segments involved (dichotomous)  ≥ 4  < 4 | 71  83 | 1.48 (1.02-2.15)  Reference | **0.04** | 1.22 (0.57-2.58) | 0.61 | 0.89 (0.55-1.43)  Reference | 0.62 | - | - |
| Pre-surgery Nr of segments involved (continuous) | 154 | 1.06 (0.96-1.18) | 0.25 | - | - | 0.93 (0.81-1.08) | 0.33 | - | - |
| Pre-surgery Nr of segments involved (dichotomous)  ≥ 4  < 4 | 50  104 | 1.26 (0.86-1.85)  Reference | 0.24 | - | - | 0.72 (0.42-1.22)  Reference | 0.23 | - | - |
| Baseline Nr of liver lesions (continuous) | 154 | 1.03 (1.01-1.06) | **0.02** | 1.03 (0.98-1.08) | 0.31 | 1.02 (0.98-1.05) | 0.35 | - | - |
| Baseline Nr of liver lesions (dichotomous)  ≥ 4  < 4 | 70  84 | 1.74 (1.19-2.53)  Reference | **0.004** | 1.58 (0.76-3.30) | 0.23 | 1.18 (0.73-1.90)  Reference | 0.50 | - | - |
| Pre-surgery Nr of liver lesions (continuous) | 154 | 1.02 (0.99-1.05) | 0.13 | - | - | 1.01 (0.97-1.04) | 0.96 | - | - |
| Pre-surgery Nr of liver lesions (dichotomous)  ≥ 4  < 4 | 56  98 | 1.48 (1.01-2.15)  Reference | **0.04** | 0.71 (0.39-1.29) | 0.26 | 0.84 (0.51-1.38)  Reference | 0.49 | - | - |
| Nr of vanished CRLMs (continuous) | 154 | 1.12 (0.98-1.28) | 0.11 | - | - | 1.16 (1.00-1.34) | **0.05** | 1.09 (0.92-1.28) | 0.34 |
| Vanished CRLMs (Dichotomous)  Yes  No | 48  106 | 0.94 (0.63-1.41)  Reference | 0.77 | - | - | 1.12 (0.67-1.87)  Reference | 0.68 | - | - |
| Baseline Max diameter of the largest liver lesion (continuous) | 154 | 1.00 (1.00-1.01) | 0.42 | - | - | 1.01 (1.00-1.01) | 0.12 | - | - |
| Pre-surgery Max diameter of the largest liver lesion (continuous) | 154 | 1.01 (1.00-1.01) | 0.17 | - | - | 1.01 (1.00-1.02) | **0.095** | 1.01 (1.00-1.02) | 0.08 |
| Baseline Nr of liver lesions in contact with vessels (continuous) | 154 | 1.09 (0.98-1.21) | 0.103 | - | - | 1.06 (0.92-1.22) | 0.43 | - | - |
| Baseline Nr of liver lesions in contact with vessels (dichotomous)  Yes  No | 93  61 | 1.34 (0.91-1.97)  Reference | 0.15 | - | - | 1.36 (0.82-2.25)  Reference | 0.23 | - | - |
| Pre-surgery Nr of liver lesions in contact with vessels (continuous) | 154 | 1.05 (0.93-1.19) | 0.45 | - | - | 0.95 (0.79-1.33) | 0.55 | - | - |
| Pre-surgery Nr of lesions in contact with vessels (dichotomous)  Yes  No | 87  67 | 1.22 (0.84-1.79)  Reference | 0.30 | - | - | 0.23 (0.75-2.00)  Reference | 0.42 | - | - |
| MRI with gadolinium-based contrast at baseline and/or before surgery  Yes  No | 101  53 | 1.10 (0.75-1.61)  Reference | 0.63 | - | - | 0.84 (0.52-1.36)  Reference | 0. 48 | - | - |
| *RAS* and *BRAF* status  *RAS* MUT  *BRAF* MUT  WT  NA | 76  4  72  2 | 0.91 (0.63-1.35)  4.03 (1.44-11.28)  Reference | 0.68  **0.008** | 1.00 (0.68-1.47)  3.50 (1.17-10.48) | 1.00  **0.03** | 1.11 (0.68-1.82)  4.50 (1.35-15.04)  Reference | 0.68  **0.01** | 1.18 (0.71-1.99)  4.88 (1.42-16.78) | 0.53  **0.01** |
| Objective response to chemotherapy  Yes  No | 108  46 | 0.87 (0.59-1.30)  Reference | 0.51 | - | - | 0.83(0.50-1.38)  Reference | 0.48 | - | - |
| Chemotherapy regimen  Triplet  Doublets | 58  96 | 0.93 (0.63-1.37)  Reference | 0.71 | - | - | 1.34 (0.82-2.16)  Reference | 0.24 | - | - |
| Biologic agent administered  Anti-EGFR  Anti-VEGF  None | 41  98  15 | 1.66 (0.81 – 3.38)  1.34 (0.69 – 2.60)  Reference | 0.17  0.39 | - | - | 1.02 (0.41-2.53)  1.12 (0.51-2.49)  Reference | 0.97  0.78 | - | - |
| Duration of chemotherapy before surgery  ≥ 3.7 months  < 3.7 months | 76  78 | 0.58 (0.40-0.84)  Reference | **0.004** | 0.70 (0.47-1.06) | 0.09 | 0.51 (0.31-0.83)  Reference | **0.007** | 0.68 (0.40-1.17) | **0.01** |
| Scheduled liver surgery  One step  Two steps | 149  5 | 0.69 (0.28-1.69)  Reference | 0.41 | - | - | 0.43 (0.13-1.37)  Reference | 0.15 | - | - |

**Legend**

CI: confidence interval; CRLM: colorectal liver metastasis; MUT: mutant; NA: not available; Nr: number; OR: odds ratio; WT: wild-type.
