# Supplementary material for: Predicting early recurrence after resection of initially unresectable colorectal liver metastases: the role of baseline and pre-surgery clinical, radiological and molecular factors in a real-life multicentre experience
Source: ESMO Open. 2024 Apr 16;9(4):102991. doi: 10.1016/j.esmoop.2024.102991 (PMC11027482; doi:10.1016/j.esmoop.2024.102991)
Supplement: Supplemental Figure 1 [file mmc3.pptx]

## Slide 1
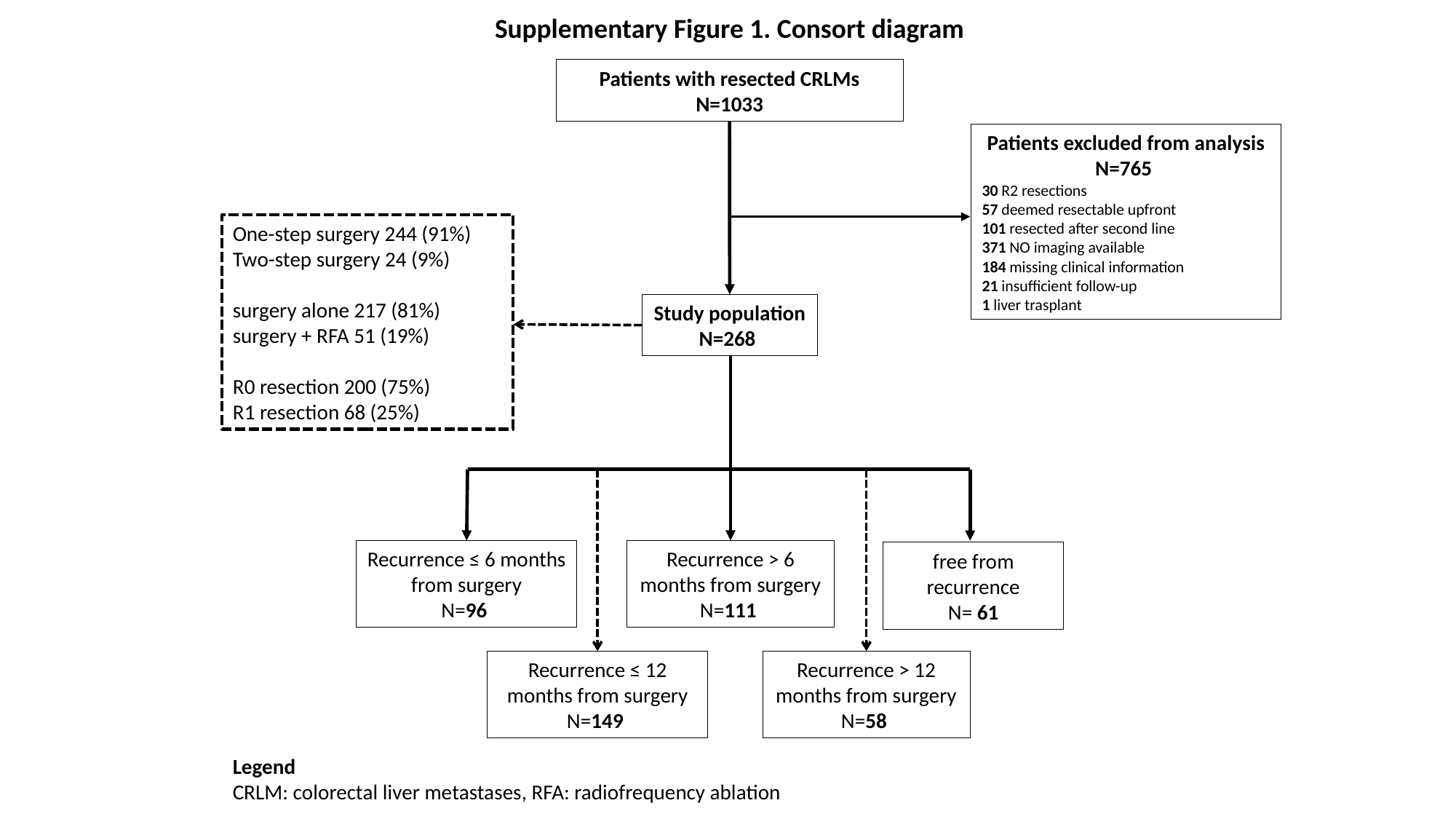

Supplementary Figure 1. Consort diagram
Patients with resected CRLMs
N=1033
Patients excluded from analysis
N=765
30 R2 resections
57 deemed resectable upfront
101 resected after second line
371 NO imaging available
184 missing clinical information
21 insufficient follow-up
1 liver trasplant
One-step surgery 244 (91%)
Two-step surgery 24 (9%)
surgery alone 217 (81%)
surgery + RFA 51 (19%)
R0 resection 200 (75%)
R1 resection 68 (25%)
Study population
N=268
Recurrence ≤ 6 months from surgery
N=96
Recurrence > 6 months from surgery
N=111
free from recurrence
N= 61
Recurrence ≤ 12 months from surgery
N=149
Recurrence > 12 months from surgery
N=58
Legend
CRLM: colorectal liver metastases, RFA: radiofrequency ablation
