# Supplementary material for: Predicting early recurrence after resection of initially unresectable colorectal liver metastases: the role of baseline and pre-surgery clinical, radiological and molecular factors in a real-life multicentre experience
Source: ESMO Open. 2024 Apr 16;9(4):102991. doi: 10.1016/j.esmoop.2024.102991 (PMC11027482; doi:10.1016/j.esmoop.2024.102991)
Supplement: Supplemental Figure 2 [file mmc4.pptx]

## Slide 1
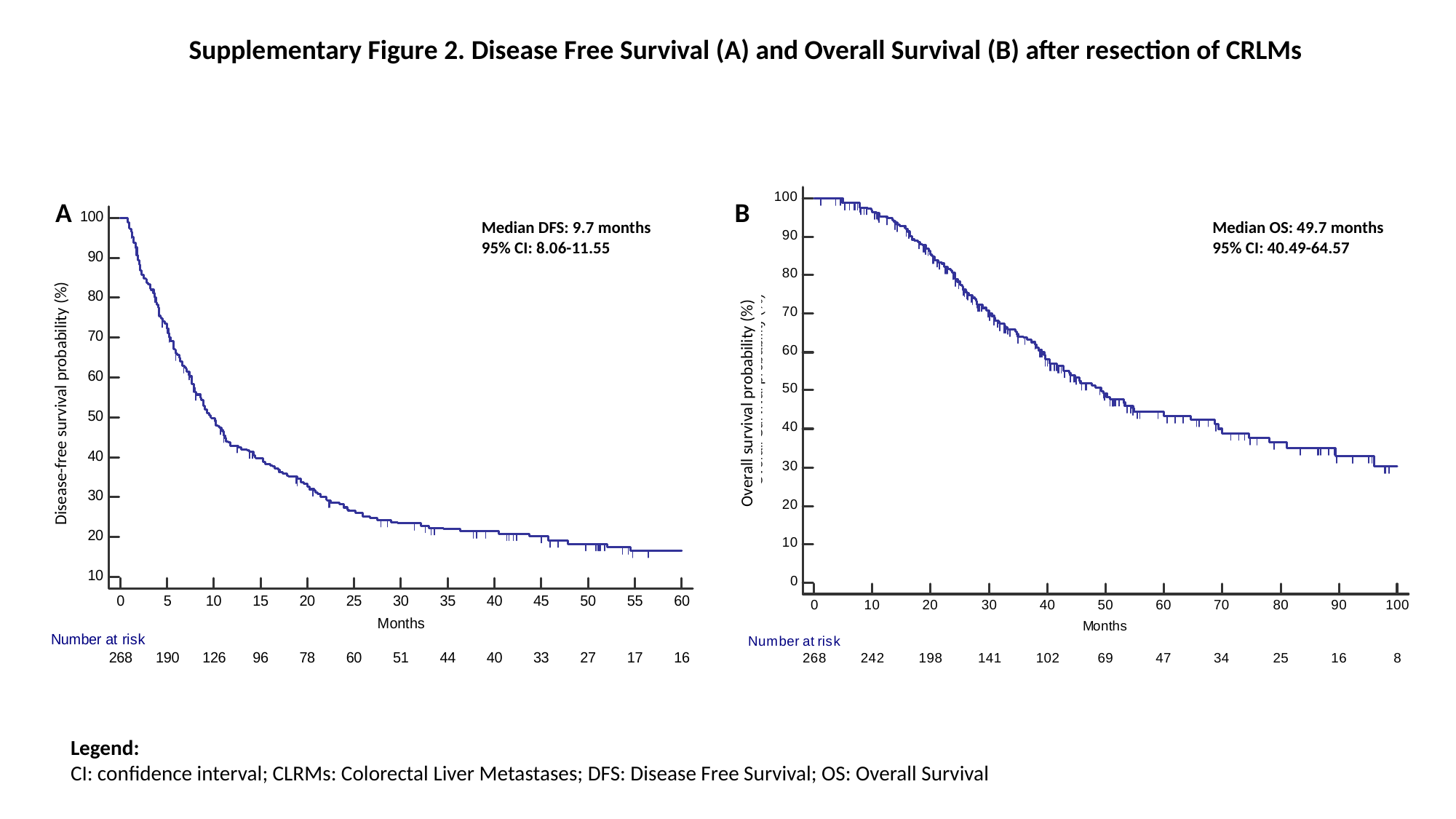

Supplementary Figure 2. Disease Free Survival (A) and Overall Survival (B) after resection of CRLMs
A
B
Median DFS: 9.7 months
95% CI: 8.06-11.55
Median OS: 49.7 months
95% CI: 40.49-64.57
Overall survival probability (%)
Disease-free survival probability (%)
Legend:
CI: confidence interval; CLRMs: Colorectal Liver Metastases; DFS: Disease Free Survival; OS: Overall Survival
