# Supplementary material for: Predicting early recurrence after resection of initially unresectable colorectal liver metastases: the role of baseline and pre-surgery clinical, radiological and molecular factors in a real-life multicentre experience
Source: ESMO Open. 2024 Apr 16;9(4):102991. doi: 10.1016/j.esmoop.2024.102991 (PMC11027482; doi:10.1016/j.esmoop.2024.102991)
Supplement: Supplemental Figure 3 [file mmc5.pptx]

## Slide 1
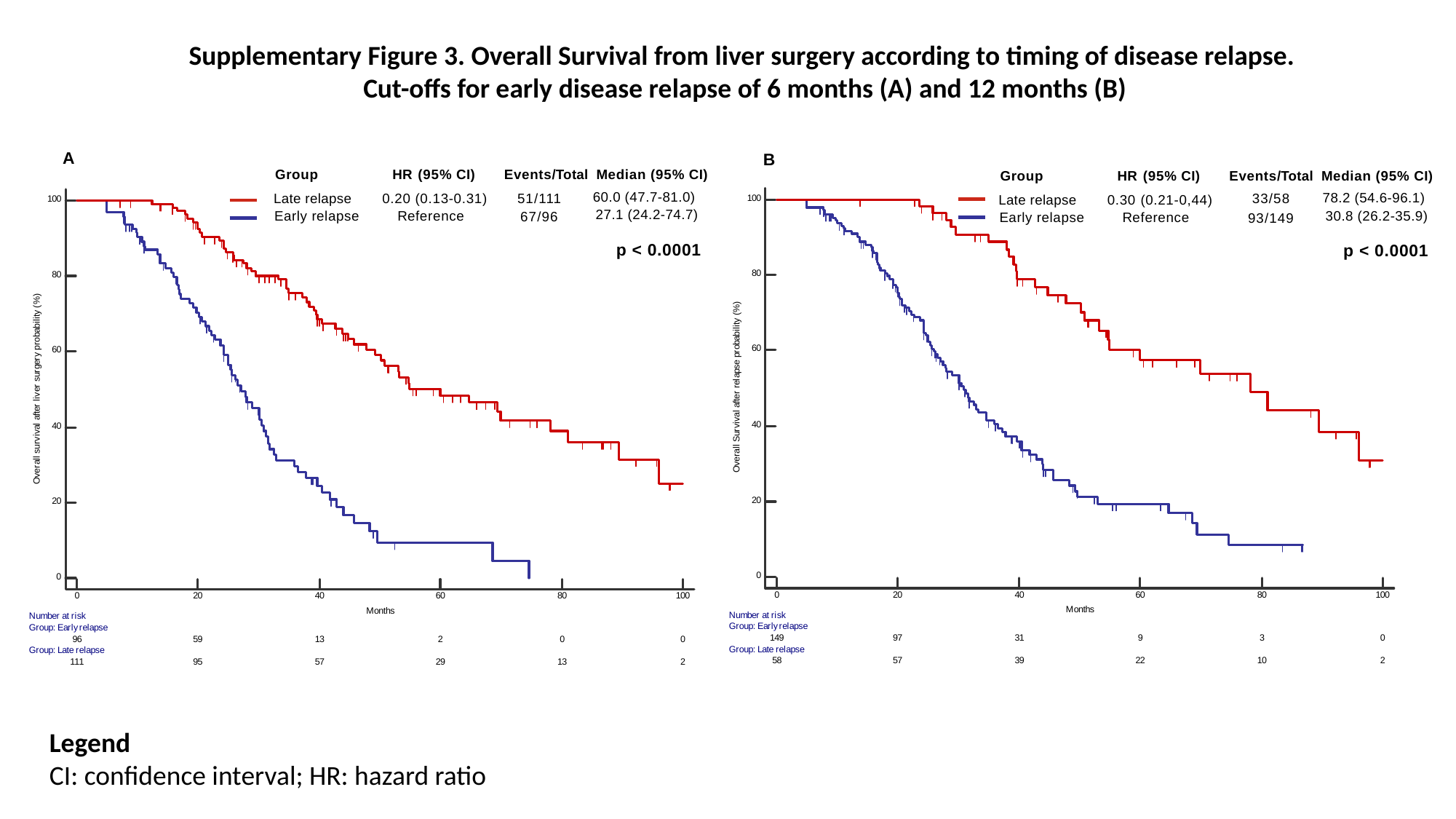

Supplementary Figure 3. Overall Survival from liver surgery according to timing of disease relapse.
Cut-offs for early disease relapse of 6 months (A) and 12 months (B)
A
B
Group HR (95% CI) Events/Total Median (95% CI)
Group HR (95% CI) Events/Total Median (95% CI)
60.0 (47.7-81.0)
27.1 (24.2-74.7)
78.2 (54.6-96.1)
30.8 (26.2-35.9)
 Late relapse 0.20 (0.13-0.31)
 Early relapse Reference
33/58
93/149
51/111
67/96
 Late relapse 0.30 (0.21-0,44)
 Early relapse Reference
p < 0.0001
p < 0.0001
Legend
CI: confidence interval; HR: hazard ratio
