# Supplementary material for: Predicting early recurrence after resection of initially unresectable colorectal liver metastases: the role of baseline and pre-surgery clinical, radiological and molecular factors in a real-life multicentre experience
Source: ESMO Open. 2024 Apr 16;9(4):102991. doi: 10.1016/j.esmoop.2024.102991 (PMC11027482; doi:10.1016/j.esmoop.2024.102991)
Supplement: Supplemental Figure 4 [file mmc6.pptx]

## Slide 1
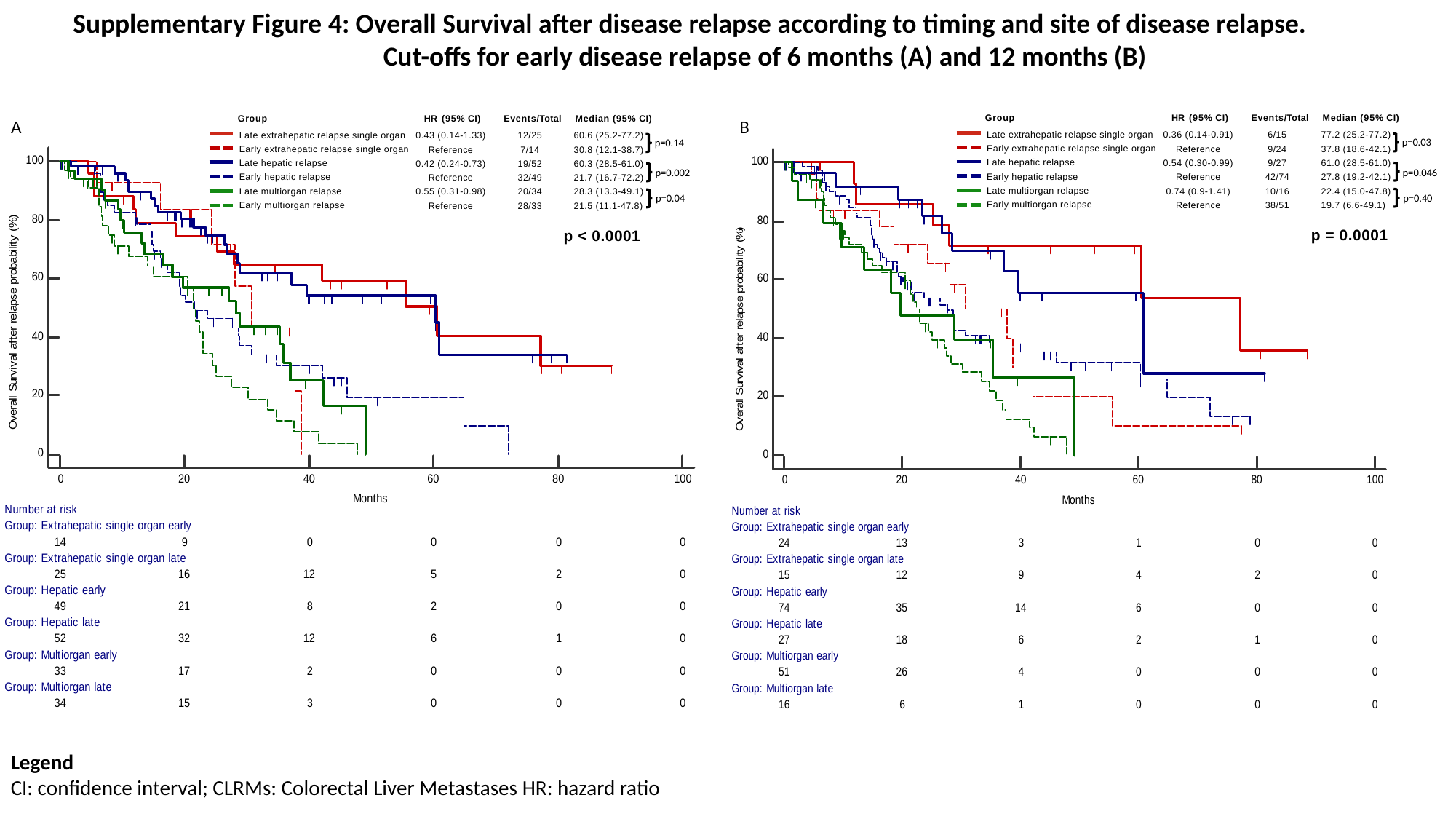

Supplementary Figure 4: Overall Survival after disease relapse according to timing and site of disease relapse.
Cut-offs for early disease relapse of 6 months (A) and 12 months (B)
B
A
Group HR (95% CI) Events/Total Median (95% CI)
Group HR (95% CI) Events/Total Median (95% CI)
Late extrahepatic relapse single organ
Early extrahepatic relapse single organ
Late hepatic relapse
Early hepatic relapse
Late multiorgan relapse
Early multiorgan relapse
0.36 (0.14-0.91)
Reference
0.54 (0.30-0.99)
Reference
0.74 (0.9-1.41)
Reference
6/15
9/24
9/27
42/74
10/16
38/51
77.2 (25.2-77.2)
37.8 (18.6-42.1)
61.0 (28.5-61.0)
27.8 (19.2-42.1)
22.4 (15.0-47.8)
19.7 (6.6-49.1)
Late extrahepatic relapse single organ
Early extrahepatic relapse single organ
Late hepatic relapse
Early hepatic relapse
Late multiorgan relapse
Early multiorgan relapse
0.43 (0.14-1.33)
Reference
0.42 (0.24-0.73)
Reference
0.55 (0.31-0.98)
Reference
12/25
7/14
19/52
32/49
20/34
28/33
60.6 (25.2-77.2)
30.8 (12.1-38.7)
60.3 (28.5-61.0)
21.7 (16.7-72.2)
28.3 (13.3-49.1)
21.5 (11.1-47.8)
p=0.03
p=0.14
p=0.046
p=0.002
p=0.40
p=0.04
p = 0.0001
p < 0.0001
Legend
CI: confidence interval; CLRMs: Colorectal Liver Metastases HR: hazard ratio
