# Supplementary material for: Predicting early recurrence after resection of initially unresectable colorectal liver metastases: the role of baseline and pre-surgery clinical, radiological and molecular factors in a real-life multicentre experience
Source: ESMO Open. 2024 Apr 16;9(4):102991. doi: 10.1016/j.esmoop.2024.102991 (PMC11027482; doi:10.1016/j.esmoop.2024.102991)
Supplement: Supplemental Figure 5 [file mmc7.pptx]

## Slide 1
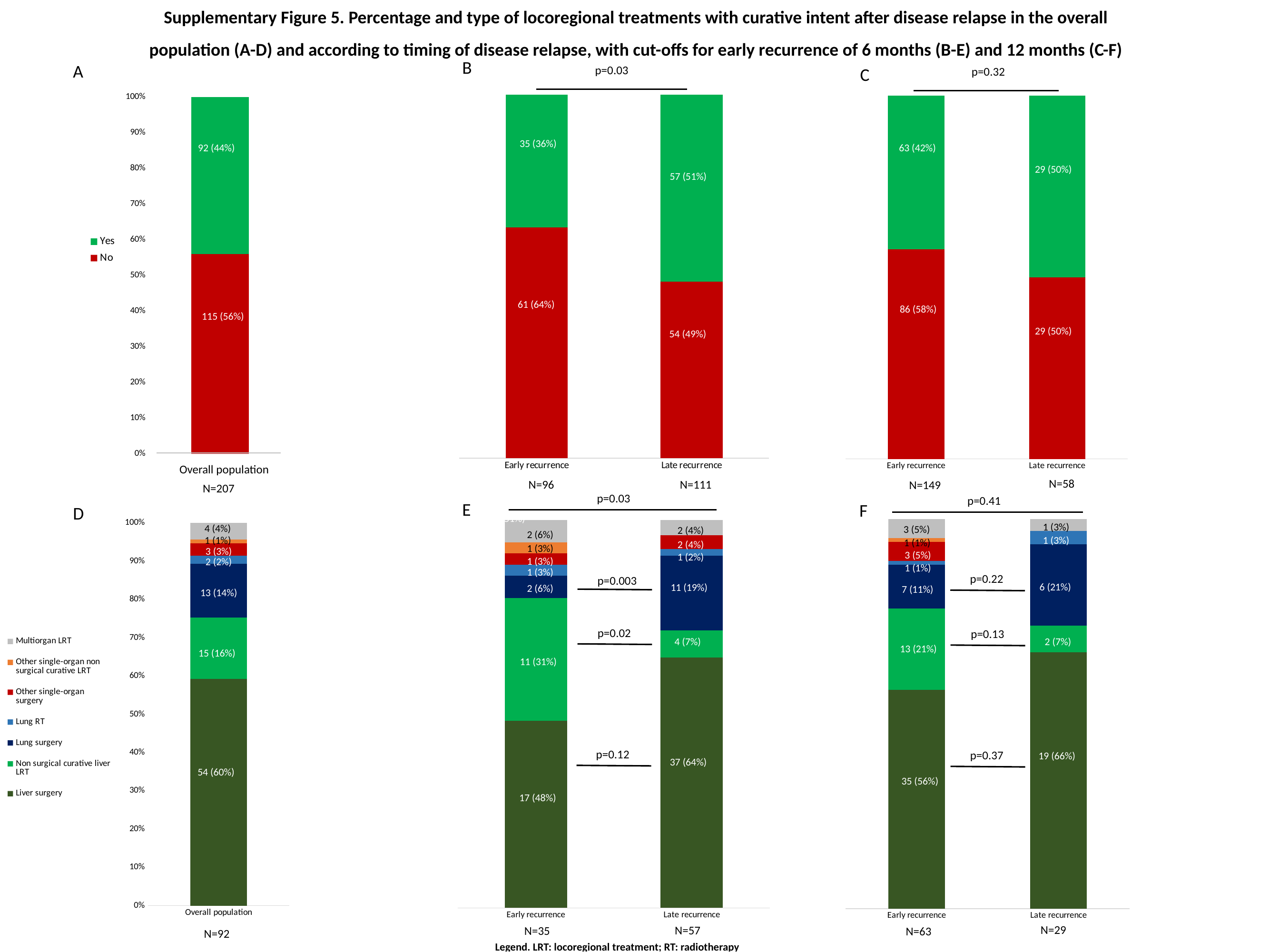

Supplementary Figure 5. Percentage and type of locoregional treatments with curative intent after disease relapse in the overall population (A-D) and according to timing of disease relapse, with cut-offs for early recurrence of 6 months (B-E) and 12 months (C-F)
B
A
p=0.03
C
p=0.32
67 (32%)
33 (34%)
34 (31%)
51 (35%)
16 (28%)
15 (26%)
14 (15%)
25 (23%)
39 (19%)
24 (16%)
Overall population
N=58
N=111
N=96
N=149
N=207
49 (51%)
101 (49%)
27 (46%)
### Chart
| Category | No | Yes |
|---|---|---|
| 1 | 0.56 | 0.44 |
### Chart
| Category | No | Yes |
|---|---|---|
| Early recurrence | 0.6354166666666666 | 0.36458333333333337 |
| Late recurrence | 0.4864864864864865 | 0.5135135135135135 |
### Chart
| Category | No | Yes |
|---|---|---|
| Early recurrence | 0.5771812080536913 | 0.42281879194630867 |
| Late recurrence | 0.5 | 0.5 |35 (36%)
92 (44%)
63 (42%)
29 (50%)
57 (51%)
61 (64%)
86 (58%)
115 (56%)
29 (50%)
54 (49%)
p=0.03
p=0.41
E
F
D
### Chart
| Category | Liver surgery | Non surgical curative liver LRT | Lung surgery | Lung RT | Other single-organ surgery | Other single-organ non surgical LRT | Multiorgan LRT |
|---|---|---|---|---|---|---|---|
| Early recurrence | 0.5555555555555556 | 0.20634920634920634 | 0.1111111111111111 | 0.01 | 0.047619047619047616 | 0.01 | 0.047619047619047616 |
| Late recurrence | 0.6551724137931034 | 0.06896551724137931 | 0.20689655172413793 | 0.034482758620689655 | 0.0 | 0.0 | 0.03 |
### Chart
| Category | Liver surgery | Non surgical curative liver LRT | Lung surgery | Lung RT | Other single-organ surgery | Other single-organ non surgical LRT | Multiorgan LRT |
|---|---|---|---|---|---|---|---|
| Early recurrence | 0.48 | 0.3142857142857143 | 0.05714285714285714 | 0.02857142857142857 | 0.02857142857142857 | 0.02857142857142857 | 0.05714285714285714 |
| Late recurrence | 0.6491228070175439 | 0.07017543859649122 | 0.19298245614035087 | 0.017543859649122806 | 0.03508771929824561 | 0.0 | 0.04 |
### Chart
| Category | Liver surgery | Non surgical curative liver LRT | Lung surgery | Lung RT | Other single-organ surgery | Other single-organ non surgical curative LRT | Multiorgan LRT |
|---|---|---|---|---|---|---|---|
| Overall population | 0.6 | 0.16304347826086957 | 0.14130434782608695 | 0.021739130434782608 | 0.03260869565217391 | 0.010869565217391304 | 0.043478260869565216 |1 (3%)
4 (4%)
3 (5%)
2 (4%)
2 (6%)
1 (1%)
1 (3%)
1 (1%)
2 (4%)
1 (3%)
3 (3%)
3 (5%)
1 (2%)
1 (3%)
2 (2%)
1 (1%)
1 (3%)
p=0.22
p=0.003
6 (21%)
11 (19%)
2 (6%)
7 (11%)
13 (14%)
p=0.02
p=0.13
4 (7%)
2 (7%)
13 (21%)
15 (16%)
11 (31%)
p=0.12
p=0.37
19 (66%)
37 (64%)
54 (60%)
35 (56%)
17 (48%)
N=29
N=57
N=35
N=63
N=92
Legend. LRT: locoregional treatment; RT: radiotherapy
