# Supplementary material for: Predicting early recurrence after resection of initially unresectable colorectal liver metastases: the role of baseline and pre-surgery clinical, radiological and molecular factors in a real-life multicentre experience
Source: ESMO Open. 2024 Apr 16;9(4):102991. doi: 10.1016/j.esmoop.2024.102991 (PMC11027482; doi:10.1016/j.esmoop.2024.102991)
Supplement: Supplemental Figure 6 [file mmc8.pptx]

## Slide 1
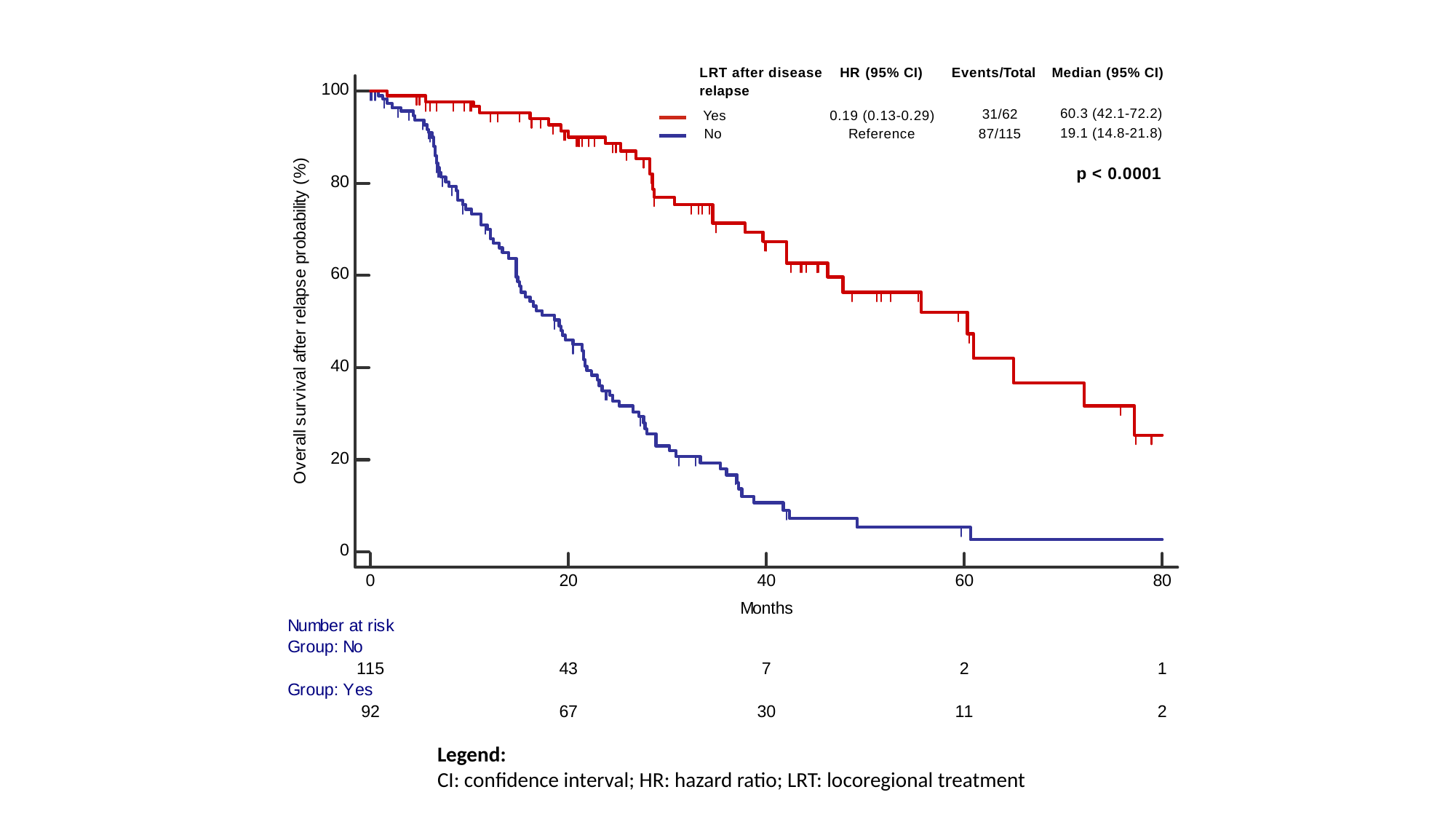

LRT after disease HR (95% CI) Events/Total Median (95% CI)
relapse
60.3 (42.1-72.2)
19.1 (14.8-21.8)
31/62
87/115
 Yes 0.19 (0.13-0.29)
 No Reference
p < 0.0001
Legend:
CI: confidence interval; HR: hazard ratio; LRT: locoregional treatment
