# Supplementary material for: Predicting early recurrence after resection of initially unresectable colorectal liver metastases: the role of baseline and pre-surgery clinical, radiological and molecular factors in a real-life multicentre experience
Source: ESMO Open. 2024 Apr 16;9(4):102991. doi: 10.1016/j.esmoop.2024.102991 (PMC11027482; doi:10.1016/j.esmoop.2024.102991)
Supplement: Supplemental Figure 7 [file mmc9.pptx]

## Slide 1
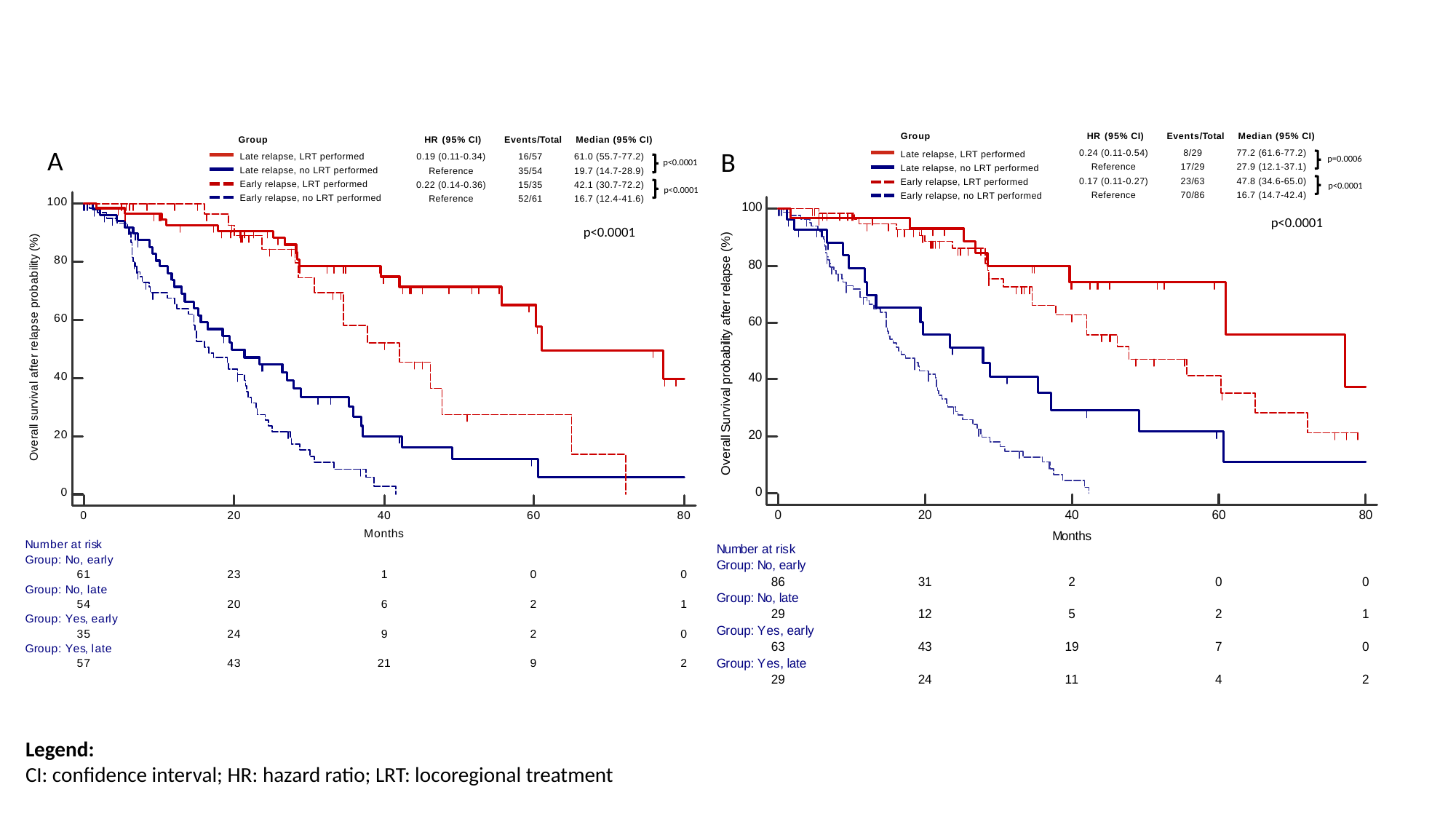

Group HR (95% CI) Events/Total Median (95% CI)
Group HR (95% CI) Events/Total Median (95% CI)
A
B
0.24 (0.11-0.54)
Reference
0.17 (0.11-0.27)
Reference
8/29
17/29
23/63
70/86
77.2 (61.6-77.2)
27.9 (12.1-37.1)
47.8 (34.6-65.0)
16.7 (14.7-42.4)
Late relapse, LRT performed
Late relapse, no LRT performed
Early relapse, LRT performed
Early relapse, no LRT performed
Late relapse, LRT performed
Late relapse, no LRT performed
Early relapse, LRT performed
Early relapse, no LRT performed
p=0.0006
0.19 (0.11-0.34)
Reference
0.22 (0.14-0.36)
Reference
16/57
35/54
15/35
52/61
61.0 (55.7-77.2)
19.7 (14.7-28.9)
42.1 (30.7-72.2)
16.7 (12.4-41.6)
p<0.0001
p<0.0001
p<0.0001
p<0.0001
p<0.0001
Legend:
CI: confidence interval; HR: hazard ratio; LRT: locoregional treatment
